# Tailoring triplet energy transfer and Förster resonance energy transfer in boron dipyrromethene modified lanthanide doped upconversion nanohybrids

**DOI:** 10.1039/d6nr02161a

**Published:** 2026-08-19

**Authors:** Weitong Xiao, Yiting Wu, Feiran Wang, Lyudmila Turyanska, Huimei Yu, Weiling Luan

**Affiliations:** a CPCIF Key Laboratory of Advanced Battery Systems and Safety, School of Mechanical and Power Engineering, East China University of Science and Technology Shanghai 200237 China luan@ecust.edu.cn; b Centre for Additive Manufacturing, Faculty of Engineering, University of Nottingham Jubilee Campus Nottingham NG8 1BB UK Lyudmila.Turyanska@nottingham.ac.uk; c School of Materials Science and Engineering, East China University of Science and Technology Shanghai 200237 China

## Abstract

Tailoring the energy transfer (ET) processes, including the singlet energy transfer (SET) or triplet energy transfer (TET) process, in hybrid systems consisting of inorganic nanoparticles and organic molecules can offer a strategy for the design and synthesis of bright fluorescent probes for photodynamic therapy or photocatalytic applications. However, the weak absorption and low quantum yield of lanthanide doped upconversion nanoparticles (UCNPs) limited the performance of the molecule–UCNP hybrid system, and the complex mechanism of the ET process involving singlet and triplet excitons is yet to be fully understood. Here, we report on a strategy for the synthesis of boron dipyrromethene (BODIPY) modified UCNP nanohybrids to engineer the SET or TET processes, specifically 8-(4-carboxyphenyl)-3,5-(4-hydroxyl)styryl-1,7-tetramethyl-pyrromethene fluoroborate (BDP-1) and 8-(4-carboxyphenyl)-2,6-diiodo-3,5-(4-hydroxyl)styryl-1,7-tetramethyl-pyrromethene fluoroborate (IBDP-1). These nanohybrids exhibit enhanced upconversion performance with an 800-times increase in upconversion quantum yield (UCQY) and efficient singlet oxygen (^1^O_2_) generation under 980 nm excitation. Our quantum chemistry calculations suggest that the energy transfer in these systems takes place by the Förster resonance energy transfer (FRET) and back energy transfer (BET) in NaYbF_4_:2%Er^3+^@BDP-1 (UCNP@BDP-1), and that direct triplet energy transfer (TET) from Yb^3+^ to anchored IBDP-1 is the dominant energy transfer mechanism in NaYbF_4_:2%Er^3+^@IBDP-1 (UCNP@IBDP-1).

## Abstract 2

Boron dipyrromethene modified upconversion nanoparticle nanohybrids are synthesised to engineer singlet or triplet energy transfer processes, leading to over 800-times increase in upconversion quantum yield and efficient singlet oxygen generation.

## Graphical abstract

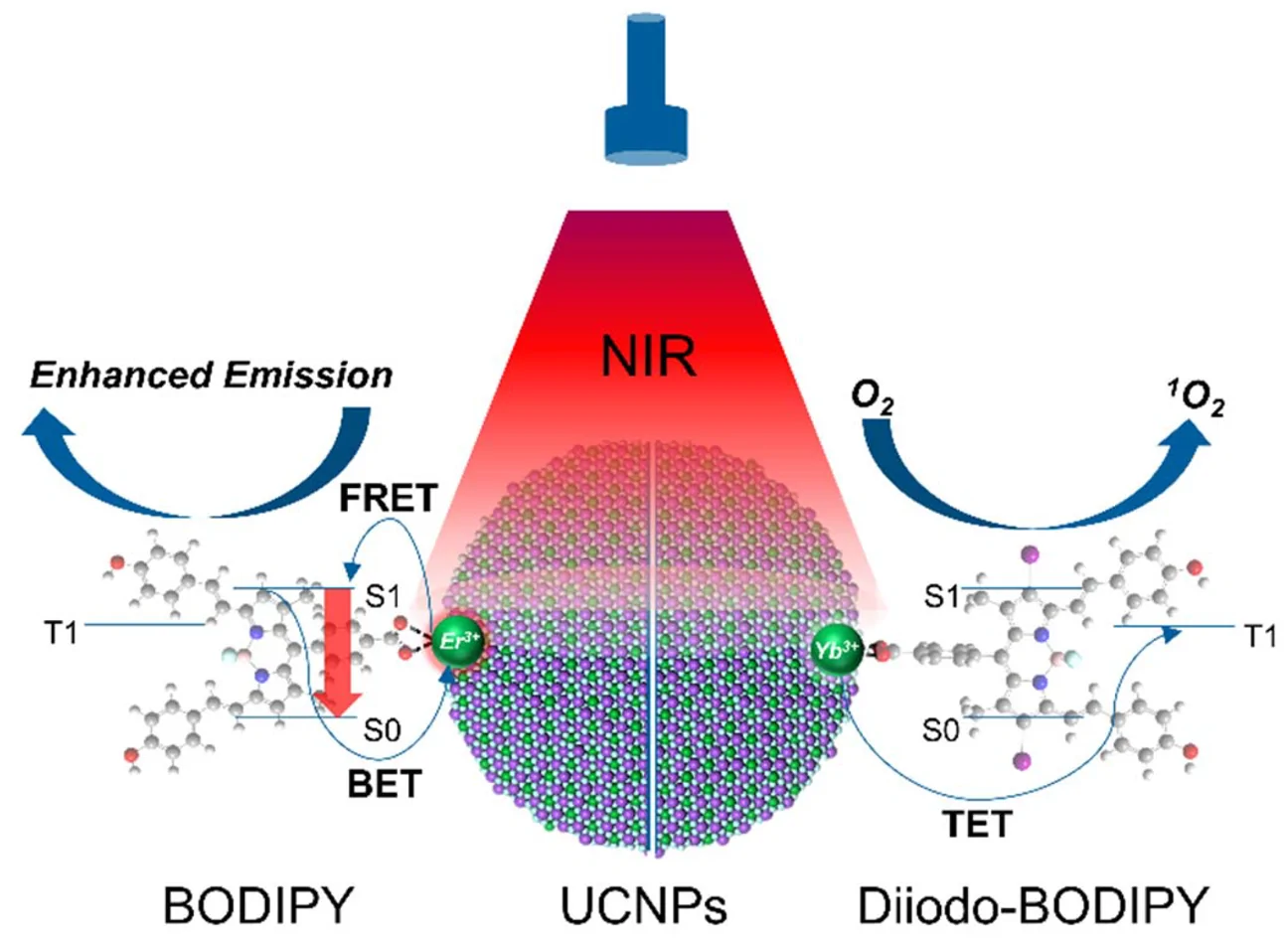

## 1. Introduction

Tailoring the energy transfer (ET) process in an organic molecular–inorganic nanoparticle hybrid system is of interest for applications, such as light harvesting,^1^ photocatalysis,^2^ probing and imaging,^3^ and photodynamic therapy (PDT).^4–7^ The hybrid organic–inorganic system not only combines the unique properties of the nanoparticle sensitizer and molecular activator, but also presents new capabilities, such as extending the wavelength range of light harvesting^8^ and generating long-lived triplet excited states or phosphorescence.^9^ The emerging reports on hybrid nano-systems based on semiconductor quantum dots (QDs),^10,11^ perovskite nanocrystals,^12^ and metal organic frameworks (MOFs)^13^ with functional organic molecules offer strategies for engineering of the singlet energy transfer (SET) or triplet energy transfer (TET) processes to explore the energy transfer mechanisms.^14^ Advantages of lanthanide doped upconversion nanoparticles (UCNPs), such as large anti-Stokes shifts, high chemical and photo-stability, and near-infrared (NIR) absorption, are well-reported.^8,15–17^ However, the construction of efficient UCNP–organic molecular nanohybrids remains challenging. Surface quenching has been extensively studied, demonstrating that energy transfer from the emissive state to high-energy vibrational states of solvent and/or ligand molecules leads to nonradiative quenching of the UCNP emission.^18–20^ On the other hand, the nature of the Laport forbidden transitions and low quantum yield of UCNPs^21–23^ result in low efficiency of the SET process from the emissive energy level of Ln^3+^ to the first excited singlet state (S_1_) of the organic component and hindering the fluorescence emission and formation of a triplet state. Construction of the core–shell structure with inert NaYF_4_ shell shielding is considered as one of the most effective strategies to block the emissive ions from the surface quenching centers and enhance the upconversion emission^21^ and is expected to enhance the SET interaction. By modifying the donor-to-acceptor separation distance, the TET process in the NaYF_4_ shell-shielded UCNP nanocomposite is highly suppressed. TET is known to require short-range Dexter-type interactions that involve both orbital overlap^24^ and spin flipping of unpaired electrons.^14,25^ Such spin-flipping capability is absent for Y^3+^ (4f^0^) but is available in the 4f orbitals of Yb^3+^ (one unpaired electron) and Er^3+^ (three unpaired electrons).^26^

In contrast, the triplet dynamics can be controlled by coupling between specific molecules and lanthanides, such as Tb^3+^, Nd^3+^, or Eu^3+^ in organic–inorganic UCNP nanohybrids.^26–28^ Effective modulation of the TET process was successfully demonstrated with an aggregation induced emission (AIE)^5^ photosensitizer and an organic triplet–triplet annihilation (TTA)^2^ photosensitizer. The organic photosensitizer can expand the absorption of the nanohybrids, and can provide a route for TET processes.^29,30^ These reports indicated that engineering of the anchored photosensitizer on the surface enabled short-range Dexter-type interactions and played an important role in the TET process,^28,29^ which in turn is essential for singlet oxygen (^1^O_2_) generation in PDT and other redox photocatalytic reactions. However, there is still no comprehensive fundamental understanding of the energy transfer mechanisms between the core–shell UCNPs and organic components. Boron dipyrromethene (BODIPY/BDP) dyes have tunable absorption and emission wavelengths, high molar extinction coefficients and fluorescence quantum yields,^31–34^ and are capable of forming the triplet state by intersystem crossing with atoms like I or Br.^35,36^ UCNP and BODIPY nanohybrids could enable ^1^O_2_ generation under NIR illumination by carefully matching the emission of a lanthanide with the absorption of BODIPY molecules,^37,38^ but the TET dominated ^1^O_2_ generation has not yet been explored.

In this work, we report on the synthesis of NaYbF_4_:2%Er^3+^@BDP nanohybrids of UCNP@BDP-1 and UCNP@IBDP-1, specifically 8-(4-carboxyphenyl)-3,5-(4-hydroxyl)styryl-1,7-tetramethyl-pyrromethene fluoroborate (BDP-1) and 8-(4-carboxyphenyl)-2,6-diiodo-3,5-(4-hydroxyl)styryl-1,7-tetramethyl-pyrromethene fluoroborate (IBDP-1). The large content of Yb^3+^ sensitizer ions in a NaYbF_4_ matrix, compared to that of the Yb^3+^-doped NaYF_4_ matrix, enhances the absorption cross-section of the nanoparticles at 980 nm, hence maximizing the utilization efficiency of excitation light and enhancing multiphoton upconversion luminescence efficiency.^16,39^

The enhanced upconversion emission and fluorescence compensation was achieved in UCNP@BDPs with an 800-times increase of the upconversion quantum yield (UCQY) observed in UCNP@IBDP-1. Both hybrid systems demonstrated the capability to generate ^1^O_2_, with effective ^1^O_2_ yield observed in the core–shell NaYbF_4_:2%Er^3+^@NaYbF_4_@IBDP-1 (YbNP@IBDP-1) as *ca.* 0.53 and the generation of ^1^O_2_ was sensitive to the IBDP-1 anchored lanthanide atom type. Our quantum chemistry calculations suggested that the energy transfer takes place by the mechanism of the Förster resonance energy transfer (FRET) and back energy transfer (BET) in UCNP@BDP-1, and direct TET from Yb^3+^ to anchored IBDP-1 dominated energy transfer in UCNP@IBDP-1. Hence, by selecting BDP molecules and lanthanide atoms in the UCNP@BDP nanohybrids, we demonstrated tailoring of the energy transfer processes and achieved enhancement of the upconversion performance or triplet generation process. These findings offer insights into energy transfer mechanisms in organic–inorganic nanohybrids, important for their applications in photodynamic therapy or other photocatalysis.

## 2. Experimental

### 2.1 Materials

All chemicals and solvents obtained from commercial sources were used without further purification. 2,4-Dimethylpyrrole (97%), 4-formylbenzoic acid (97%), 2,3-dichloro-5,6-dicyano-*p*-benzoquinone (DDQ, 98%), triethylamine (TEA, 99%), boron trifluoride diethyl etherate (BF_3_·OEt_2_, >46.5%), 4-hydroxybenzaldehyde (98%), oleic acid (OA, 90%), 1-octadecene (ODE, 90%), and 1,3-diphenylisobenzofuran (DPBF) were purchased from Sigma Aldrich. Trifluoroacetic acid (TFA, 99%), iodine (99%), iodic acid (99.5%), yttrium(iii) chloride hexahydrate (YCl_3_·6H_2_O, 99.9%), ytterbium(iii) chloride hexahydrate (YbCl_3_·6H_2_O, 99.9%), and erbium(iii) chloride hydrate (ErCl_3_·6H_2_O, 99.9%) were purchased from Aladdin (Shanghai, China). Ammonium fluoride (NH_4_F, ≥98%), sodium hydroxide (NaOH), ethanol absolute (EtOH), sodium thiosulfate (Na_2_S_2_O_3_, 99%), dichloromethane anhydrous (DCM, 98+%), methanol (MeOH), *n*-hexane and sodium sulfate anhydrous (Na_2_SO_4_, >99%) were purchased from General Reagent.

### 2.2 Synthesis of Ben-BDP

Ben-BDP was synthesized by following a reported procedure.^12^ Briefly, 4-formylbenzoic acid (160 mg, 1 mmol, 1 equiv.) was pre-dissolved in 20 ml dried dichloromethane. Subsequently, 2,4-dimethyl pyrrole (260 μL, 2.5 mmol, 2 equiv.) and drops of trifluoroacetic acid were added. The mixture was left stirring at room temperature under argon for 6 h. Then, a mixture solution of 2,3-dichloro-5,6-dicyano-*p*-benzoquinone (227 mg, 1 mmol) and 20 ml dichloromethane was added to the reaction mixture and reacted for 1 h. After that, triethylamine (2 ml, 16 mmol, 16 equiv.) was added followed by dropwise addition of BF_3_·OEt_2_ (2 ml, 16 mmol, 16 equiv.). The reaction was stopped after 12 h reaction and the solvent was evaporated under reduced pressure. The obtained reaction crude product was subjected to silica gel column chromatography and purified (DCM : MeOH = 30 : 1, v/v) to give a purple-red solid (100 mg, 27% yield): ^1^H NMR (400 MHz, DMSO-*d*6) *δ* 8.10 (d, *J* = 8 Hz, 2H), 7.53 (d, *J* = 8 Hz, 2H), 6.20 (s, 2H), 2.46 (s, 6H), 1.33 (s, 6H) ppm. ^13^C NMR (101 MHz, DMSO-*d*6) *δ* 166.84, 155.29, 142.65, 140.92, 140.77, 138.42, 132.18, 130.15, 128.41, 121.60, 14.23, 14.05 ppm. HRMS (ESI) for C_20_H_19_BF_2_N_2_O_2_H^+^ [M + H]^+^: 369.1988, found: 369.1583.

### 2.3 Synthesis of BDP-1

The obtained Ben-BDP (50 mg, 0.14 mmol, 1 equiv.) and 4-hydroxybenzaldehyde (40 mg, 0.36 mmol, 2.5 equiv.) were dissolved in 30 ml dried toluene. Then 150 μL piperidine and 150 μL glacial acetic acid were added. With the protection of an argon atmosphere, the reaction was carried out at 130 °C with stirring and refluxing overnight. The solid residue was subjected to silica gel column chromatography and eluted (DCM : methanol = 90 : 10, v/v) to give a dark brown solid (18 mg, 21% yield): ^1^H NMR (400 MHz, DMSO-*d*6) *δ* 8.10–8.12 (d, *J* = 8 Hz, 2H), 7.58–7.60 (d, *J* = 8 Hz, 2H), 7.47–7.51 (d, *J* = 16 Hz, 2H), 7.47–7.49 (d, *J* = 8 Hz, 4H), 7.33–7.37 (d, *J* = 16 Hz, 4H), 6.94 (s, 2H), 6.86–6.88 (d, *J* = 8 Hz, 4H), 1.39 (s, 6H) ppm. ^13^C NMR (101 MHz, DMSO-*d*6) *δ* 172.02, 166.33, 159.10, 152.52, 141.05, 138.86, 137.32, 135.83, 131.94, 130.02, 129.55, 129.10, 127.31, 118.15, 116.13, 115.29, 14.32 ppm. HRMS (ESI) for C_34_H_27_BF_2_N_2_O_4_H^+^ [M + H]^+^: 577.4151, found: 577.2107.

### 2.4 Synthesis of IBDP

The obtained Ben-BDP (47.7 mg, 0.13 mmol, 1 equiv.) and iodine (81.5 mg, 0.32 mmol, 2.5 equiv.) were dissolved in 20 ml ethanol. Then 1 ml iodic acid (45.7 mg, 0.26 mmol, 2 equiv.) water solution was added dropwise. The reaction was performed at 60 °C under stirring for 1 h. The reaction crude product was mixed with saturated Na_2_S_2_O_3_ solution under reduced pressure to remove the ethanol. Then, the residue was extracted with DCM three times and the organic components were dried over the anhydrous Na_2_SO_4_. The products were subjected to silica gel column chromatography and eluted (DCM : methanol = 30 : 1, v/v) to give a red solid (26.6 mg, 33% yield): ^1^H NMR (400 MHz, DMSO-*d*6) *δ* 8.11–8.13 (d, *J* = 8 Hz, 2H), 7.56–7.58 (d, *J* = 8 Hz, 2H), 2.55 (s, 6H), 1.33 (s, 6H) ppm. ^13^C NMR (101 MHz, DMSO-*d*6) *δ* 166.76, 156.45, 144.83, 140.61, 138.18, 131.90, 130.43, 130.35, 128.42, 87.17, 16.66, 15.82 ppm. HRMS (ESI) for C_20_H_17_BN_2_O_2_F_2_I_2_H^−^ [M − H]^−^: 618.9760, found: 618.9379.

### 2.5 Synthesis of IBDP-1

The obtained IBDP (116.0 mg, 0.14 mmol, 1 equiv.) and 4-hydroxybenzaldehyde (40 mg, 0.36 mmol, 2.5 equiv.) were dissolved in 15 ml dried toluene. Then 150 μL piperidine and 150 μL glacial acetic acid were added. With the protection of an argon atmosphere, the reaction was performed at 130 °C under stirring and refluxing for 2 h. The sold residue was subjected to silica gel column chromatography and eluted (DCM : methanol = 20 : 10, v/v) to give a dark brown solid (67 mg, 69% yield): ^1^H NMR (400 MHz, DMSO-*d*6) *δ* 8.14–8.12 (d, *J* = 8 Hz, 2H), 8.09–8.04 (d, *J* = 20 Hz, 2H), 7.62–7.60 (d, *J* = 8 Hz, 2H), 7.49–7.47 (d, *J* = 8 Hz, 4H), 7.36–7.41 (d, *J* = 20 Hz, 2H), 6.90–6.88 (d, *J* = 8 Hz, 4H), 1.39 (s, 6H) ppm. HRMS (ESI) for C_34_H_25_BN_2_O_4_F_2_I_2_Na^+^ [M + Na]^+^: 851.1900, found: 850.9856.

### 2.6 Synthesis of the UCNPs and core–shell UCNPs

NaYbF_4_:2%Er^3+^ nanoparticles were synthesized following a reported protocol^40^ with some modifications in the addition of Na^+^ and F^−^ precursors in this work. Briefly, 379.8 mg YbCl_3_·6H_2_O (0.98 mmol) and 7.6 mg ErCl_3_·6H_2_O (0.02 mmol) solid were combined with 10 ml oleic acid (OA) and 20 ml 1-octadecene (ODE) in a 100 ml three neck round bottom flask. The solution was stirred under an Ar atmosphere at temperature *T* = 160 °C for 1 h. After cooling to 50 °C, for the *In situ* Mixing (IM) protocol as the traditional protocol indicated, 5 ml NaOH (0.5 M) methanol solution and 16 ml NH_4_F (0.5 M) methanol solution were added dropwise into the flask and stirred for 1 h until the solution became clear. For the Separative Addition (SA) protocol, 100 mg NaOH (2.5 mmol) solid was added to the mixture and heated to 160 °C for 1 h, and after cooling to 50 °C again, 16 ml NH_4_F (0.5 M) methanol solution was added and held for 1 h to evaporate methanol. Then the solution was degassed at *T* = 110 °C for 15 min to remove the residual oxygen, water, and methanol. The solution was heated to 315 °C for 1 h under continuous stirring. After cooling to room temperature, the nanoparticles were precipitated with ethanol, and collected by centrifugation at 6000 rpm for 10 min and washed three times with hexane/ethanol (1/4 v/v) solution and centrifugation. The final product was dried and the collected powder was stored at room temperature under ambient atmosphere.

The synthesis of NaYbF_4_:2%Er^3+^@NaYbF_4_ nanoparticles followed the hot-injection protocols.^41^ Briefly, a 0.5 mmol shell precursor was injected into a fresh solvent at 315 °C (10.5 ml OA + 10.5 ml ODE) containing 1 mmol core NaYbF_4_. The NaYbF_4_ shell and NaErF_4_ shell precursor were prepared by the SA protocol with the corresponding salt.

### 2.7 Synthesis of UCNPs@BODIPY nanohybrids

A UCNPs@BODIPY hybrid system was prepared based a modified published protocol.^42^ Briefly, OA capped UCNPs were precipitated by adding ethanol and centrifuged. Then, 10 mg UCNPs were redissolved in 5 ml chloroform and a DMSO solution of 10 nmol diluted BDPs was added, specifically 29 μL BDP-1 (0.346 mM) and 43 μL IBDP-1 (0.235 mM), respectively. The molar ratio of UCNPs/BDPs was calculated as 1 : 24.7 based on the TEM size and crystal structure of the nanoparticles. The mixture was sonicated for 30 min and left to stand still for 1 h. Then the mixture was kept under magnetic stirring overnight. The final products were washed by adding ethanol and centrifuged at 6000 rpm for 10 min. The precipitate was redispersed in DMSO and stored at 4 °C for further experiment.

### 2.8 Triplet quantum yield measurement

The triplet quantum yields of Rose Bengal (RB) and UCNP@IBDP-1 were measured by an indirect method of recording the singlet oxygen (^1^O_2_) induced absorption decay of 1,3-diphenylisobenzofuran (DPBF).^13,43^ The concentration of DPBF in 3 ml DMSO for each measurement was fixed to 50 μM. For the measurement, the cuvette with solution containing DPBF and photosensitizer was placed into a dark box and irradiated for 20 s at fixed time intervals. For the reference control, Rose Bengal (2.5 μM) was irradiated with a 540 nm (3 W) torch, and the nanohybrid was irradiated with a 980 nm laser (2 W). The absorption of the solution was recorded using a UV-Vis spectrophotometer (PerkinElmer Lambda 950).

### 2.9 Quantum chemistry calculations

Density functional theory (DFT) and time-dependent DFT (TD-DFT) calculations were carried out using Gaussian 16W. The B3LYP functional and the standard basis set 6-311G(d,p) were employed for C, H, O, B, F and N elements. The Lanl2DZ basis set was assigned to I element. Grimme's D3BJ dispersion correction was performed and orbital maps were obtained using GaussView 6.0.

### 2.10 Characterization methods

Transmission electron microscopy (TEM) was carried out on a JEOL JEM 2100 Plus (Japan), *d*-spacing of the high-resolution images of nanoparticles was determined using DigitalMicrograph, and images were analyzed using ImageJ. Powder X-ray Diffraction (XRD) patterns were recorded on a Rigaku Ultimate IV powder X-ray Cu Kα radiation diffractometer (Japan) using Cu Kα radiation (*λ* = 0.1548 nm). The upconversion spectra and emission decay were recorded on Edinburgh FLS1000 and FS5 with excitation provided by a 980 nm continuous wave laser diode (MDL-III-2W, China). PL decay curves were recorded with a 40 μs excitation pulse width and a 50 Hz source frequency of the laser, the decay results were fitted with the double-exponential function *I*(*t*) = *A*_1_e^−*t*/*τ*_1_^ + *A*_2_e^−*t*/*τ*_2_^ to analyze the raw decay curves and the average lifetime was calculated using *τ*_average_ = (*A*_1_*τ*_1_^2^ + *A*_2_*τ*_2_^2^)/(*A*_1_*τ*_1_ + *A*_2_*τ*_2_). The rise times of the emissive state in the decay spectra were described as the time interval for the upconversion luminescence intensity to increase from 10% to 90% of its maximum value.

The absolute photoluminescence quantum yields (*Φ*) were measured for all samples on an FLS1000 photoluminescence spectrometer equipped with an integrating sphere system (diameter: 150 mm); the blank solvents were used as the reference and calculated as QY = *N*_em_(*λ*_ex_)/*N*_abs_(*λ*_ex_) = (*L*_sample_ − *L*_reference_)/(*E*_reference_ − *E*_sample_), where *L* represents the integration of the emission spectrum and *E* represents the integration of the excitation spectrum. The QY of the synthesized molecules was measured using a xenon light source, and the upconversion QYs of UCNPs and nanohybrids were measured using a 980 nm laser diode (MDL-III-2W, China).

The ^1^H (400 MHz) and ^13^C (101 MHz) nuclear magnetic resonance (NMR) spectra were recorded on a Bruker AVANCE III. High-resolution mass spectrometry (MS) was performed using an Agilent 1290-6545XT (USA), electrospray ionization (ESI) was used to generate ions and methanol was used as solvent. The fluorescence spectra were recorded on the PerkinElmer LS-55 Spectrometers and the UV-vis absorption spectra were recorded on a PerkinElmer Lambda 950 spectrophotometer. Fourier transform infrared spectroscopy (FTIR) was performed on a Thermo Fisher Scientific Nicolet iS20 (USA). Dynamic light scattering (DLS) and zeta potential measurement were performed on a Malvern Zetasizer Nano ZS90 (UK).

## 3. Results and discussion

**Fig. 1:**
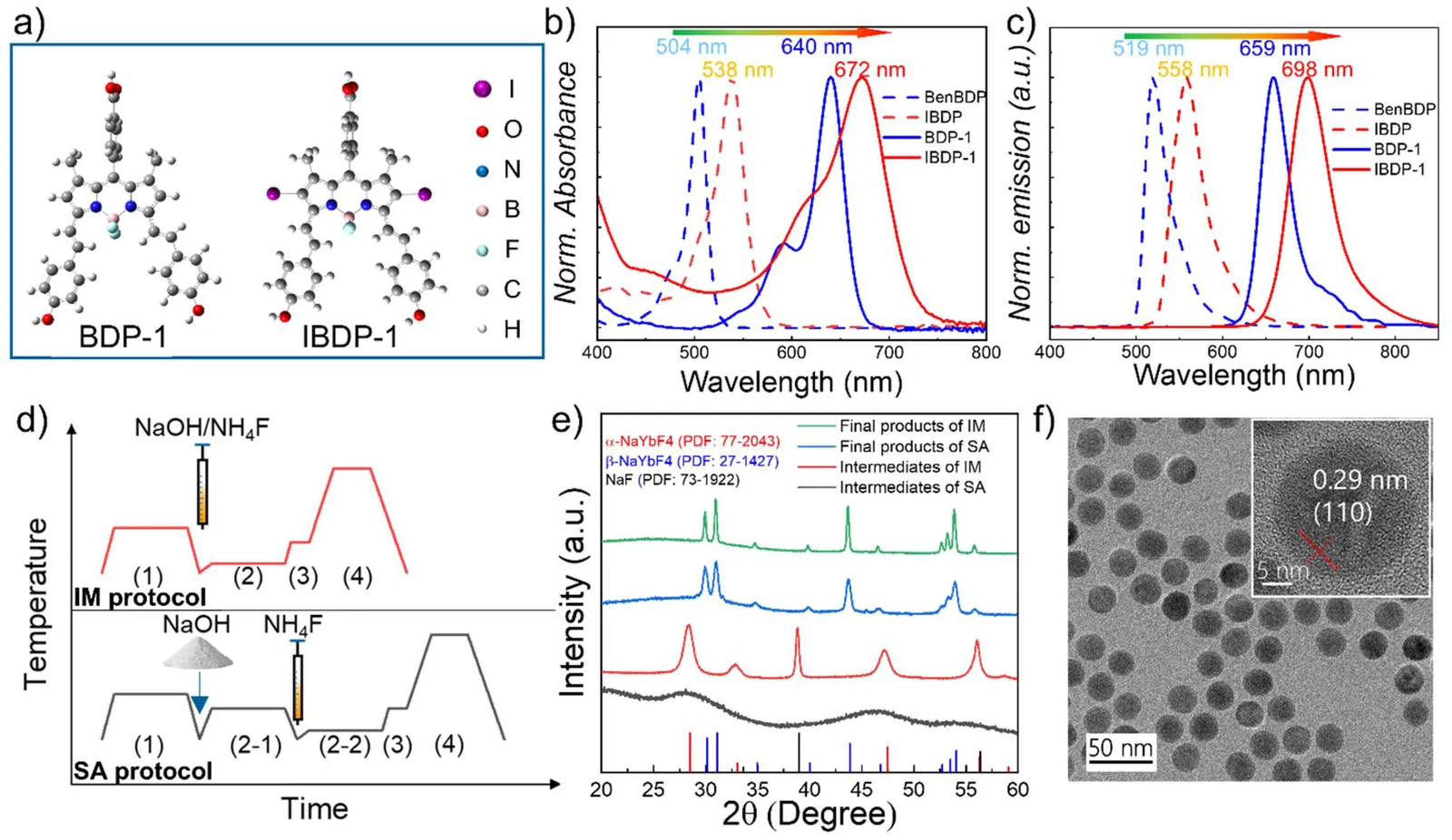
(a) Chemical structure of boron dipyrromethene (BDP) molecules used to sensitize UCNPs and their (b) absorbance and (c) fluorescence emission spectra. (d) Schematic diagram of the *in situ* mixing (IM) and separative addition (SA) protocols used for UCNP synthesis, in which the stage (1) represents the formation of the oleic acid–lanthanide complex, state (2) represents the reaction of the complex with a Na^+^ and F^−^ precursor, stage (3) is the cleaning procedure before the final reaction, and stage (4) represents the high temperature reaction in solvents. (e) XRD spectra of the intermediates and final products produced using SA and IM protocols. (f) Representative TEM and (inset) high-resolution HR-TEM images of the OA-capped UCNPs obtained by the SA protocol.

To produce a donor–acceptor UCNP-BODIPY hybrid nano-system, 8-(4-carboxyphenyl)-3,5-(4-hydroxyl)styryl-1,7-tetramethyl-pyrromethene fluoroborate (BDP-1) and 8-(4-carboxyphenyl)-2,6-diiodo-3,5-(4-hydroxyl)styryl-1,7-tetramethyl-pyrromethene fluoroborate (IBDP-1) molecules were synthesized (Scheme S1), following the reported procedures^12,44^ through the piperidine catalyzed Knoevenagel reaction.^45^ The structure of the BDPs was confirmed by ^1^H-NMR, ^13^C-NMR and HRMS (Fig. 1a and Fig. S1–S4). These measurements also indicated the presence of impurities (solvents (DMSO) and polymerization products/reagents); however these do not affect the reaction and are removed during the NP purification steps. We observed a red shift of the absorption and emission of final products from the visible to the first near-infrared window of low absorption of biological tissues (∼700–950 nm) (Fig. 1b and c),^46^ consistent with the predicted performance expected from extending the conjugation *via* specific groups (4-hydroxybenzaldehyde) and introducing heavy atoms (iodine) into the core of the BenBDP and IBDP molecules.^33^

The obtained BDP-1 and IBDP-1 have high molar extinction coefficients, *ε* = 39 000 M^−1^ cm^−1^ and 21 000 M^−1^ cm^−1^, with maximum absorbance peaks at 659 nm and 672 nm (Table 1), respectively. Importantly, we achieved highly matched absorption of BDP-1 and IBDP-1 to the energy gap of ^4^F_9/2_ → ^4^I_15/2_ (655 nm) in Er^3+^. Optical studies revealed a small Stokes shift between the emission and excitation wavelengths (Fig. 1c), and the absolute photoluminescence quantum yields *Φ* = 52.2% and 25.2% (Table 1) for BDP-1 and IBDP-1, respectively.

**Table 1:** Optical and photochemical properties of BDPs in DMSO solution

	Absorption peak position, *λ*_em_/nm	Emission peak position, *λ*_em_/nm	*ε*/10^4^ M^−1^ cm^−1^	*Φ*/%	*εΦ*/10^4^ M^−1^ cm^−1^
BDP-1	640	659	5.3	52.2	2.8
IBDP-1	672	689	2.1	25.2	0.53

As the donor components of the hybrid nano-system, colloidal Er^3+^ doped NaYbF_4_ upconversion nanoparticles, UCNPs (NaYbF_4_:2%Er^3+^) were synthesized by a separative addition (SA) protocol, in which the Na^+^ precursor and F^−^ precursor were sequentially and individually added, different from the traditional *in situ* mixing (IM) protocol^40,41,47^ (Fig. 1d). The UCNP had a hexagonal phase (Fig. 1e and Fig. S6a), and the formed spherical nanoparticles with an average diameter of 22.8 ± 1.1 nm obtained by the SA protocol were much smaller than the 62.2 ± 3.0 nm nanoparticles obtained by the IM protocol (Fig. 1f and Fig. S6b). The XRD analysis of the intermediates of the two protocols, in which the intermediates were extracted and purified before heating the solvent to 315 °C, suggested that the separative addition of the Na^+^ precursor and F^−^ precursor resulted in blocking the formation of cubic NaYbF_4_ (Fig. 1e and Fig. S6c), which facilitated the delayed nucleation and growth process of hexagonal NaYbF_4_ in high temperature solvent.^48,49^

**Fig. 2:**
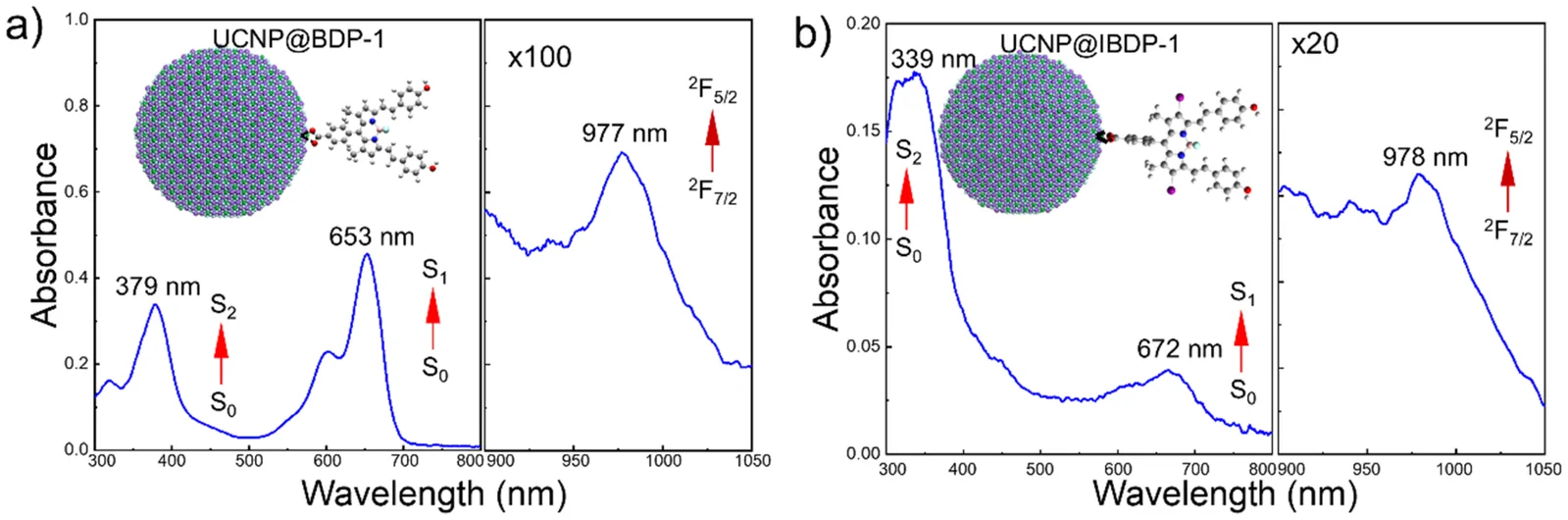
Absorption spectra and (insets) schematic illustrations of the (a) as-synthesized UCNP@BDP-1 and (b) UCNP@IBDP-1 in DMSO solutions. All samples were used with a concentration of 0.01 mg ml^−1^.

The UCNP@BDP nanohybrids were prepared through a ligand exchange procedure.^42^ Briefly, oleic acid capped UCNPs were dispersed in chloroform and stirred with BDP overnight. The BDP was exchanged with oleic acid by the carboxy group at the *meso* position and it was anchored to the lanthanide atoms at the surface of UCNPs.^42^ We note that the presence of impurities does not affect the synthesis of the UCNPs@BDP nanohybrid, and they are removed during NP purification steps. Fourier transform infrared (FT-IR) spectroscopy revealed characteristic peaks of both OA and BDP, hence confirming partial replacement of the ligands, and the formation of UCNP@BDPs and BDPs (Fig. S7). Dynamic light scattering (DLS) measurements performed in aqueous solution (1 mg ml^−1^) revealed that the hydrodynamic sizes of UCNP@BDP-1 and UCNP@IBDPs are 240.9 ± 14.4 nm and 230.7 ± 11.8 nm, respectively (Fig. S8), which are larger than those of the original UCNPs: 22.8 ± 1.1 nm size as measured by TEM. These results suggest that incomplete ligand exchange forms a complex surface with both BDP and OA present, which can induce nanoparticle aggregation in aqueous solution. Zeta potential measurements revealed values of −9.3 ± 0.6 mV and −20.7 ± 0.9 mV for UCNP@BDP-1 and UCNP@IBDP-1, respectively, in contrast to the positive zeta potential values measured for ligand-free UCNPs (+19.1 ± 0.1 mV). The formation of the UCNP@BDP nanohybrids modified the absorption profiles of UCNPs (NIR absorption peak at ∼980 nm) and BDP molecules (Fig. 2 and Fig. S5), which reveal a distinct absorption peak in the visible and NIR regions, corresponding to BDP (S_0_ → S_1_, S_0_ → S_2_) and Yb^3+^ (^2^F_7/2_ → ^2^F_5/2_) transitions, respectively. The ratio of the first absorption/second absorption also decreased significantly. We attributed the modified absorption profiles to the orbital coupling of the 4f orbitals in Er^3+^/Yb^3+^ to the frontier molecular orbitals in BDP molecules.

**Fig. 3:**
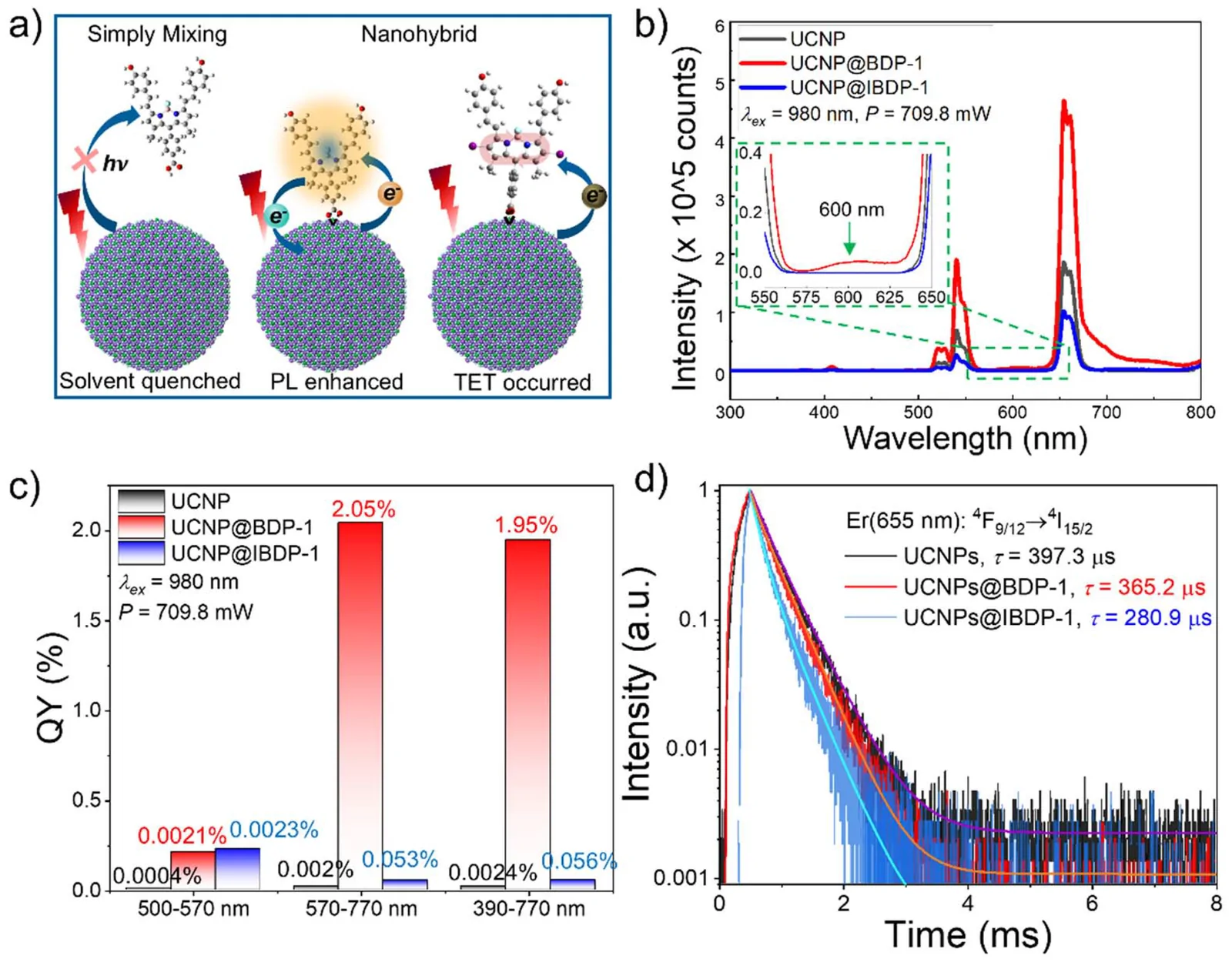
(a) Schematic illustration of the energy transfer process in the mixture of the UCNP and BDP system, UCNP@BDP-1 nanohybrid and UCNP@IBDP-1 nanohybrid. (b) Upconversion spectra of UCNP@BDPs (same concentration of 1 mg ml^−1^ in DMSO). (c) Upconversion quantum yields of UCNP@BDPs estimated using an integrating sphere method and (d) upconversion emission decay measured for the peak centred at *λ*_em_ = 655 nm. Note: all the UCNPs are NaYbF_4_:2%Er^3+^.

Upconversion emission measurements of UCNP@BDPs were performed to verify the energy transfer between the UCNP (donor) and BDPs (acceptor) under 980 nm illumination. In these donor–acceptor pairs, the red emissive states of Er^3+^ in NaYbF_4_:2%Er^3+^ UCNPs overlapped with the absorption of the BDP-1 and IBDP-1 molecules (Table 1). The upconversion spectra of the as-synthesized UCNP and the BDP mixture revealed that BDPs induced quenching of the red emission band of the UCNP (Fig. 3a and Fig. S9a), likely due to radiative energy transfer between the UCNPs and BDPs in solution, whereby under excitation at 980 nm the UCNPs (donor) emit ‘red’ photons which can be absorbed by the BDPs (acceptor) in the mixture, resulting in new emission features (Fig. S9a). Interestingly, the emission profiles of the UCNP@BDP nanohybrids revealed different features under 980 nm excitation (Fig. 3b). The upconversion emission at the red region of UCNP@BDPs was retained, in which a small peak centred at *λ*_em_ = 600 nm and a long emission tail after 670 nm appeared in UCNP@BDP-1 (Fig. 3b and Fig. S10b). Note that the emission tail intensity scales with the used excitation power (Fig. S10), and hence it is likely to be associated with the fluorescence compensation of the surface anchored BDPs (S_1_ → S_0_), which is affected by orbital coupling of ^4^F_9/2_ of Er^3+^ to the S_1_ state of BDPs. The enhanced upconversion emission of UCNP@BDP-1 could be attributed to the reduced nonradiative cross-relaxing in the UCNPs, BDP-1 anchored on the surface introduced the FRET and backward energy transfer (BET) routes and more electrons were able to populate the emissive energy level of Er^3+^ (Fig. 3a).^39^ The fluorescence compensation phenomenon of a new peak centered at 720 nm was also observed in UCNP@IBDP-1 (Fig. 3b and Fig. S10c); however the overall upconversion emission intensity decreased, suggesting a new energy transfer route and suppression of the FRET and BET processes.

The upconversion quantum yield (UCQY) results also confirmed that the BDPs induced an enhancement of upconversion emission. The UCNPs without the protection of the NaYF_4_ inert shell have UCQY ∼ 10^−5^,^22^ and the UCQY increased 20-times and 800-times in the wavelength range of 390–770 nm for UCNP@IBDP-1 and UCNP@BDP-1, respectively (Fig. 3c). This was mostly due to an increase of emission at *λ*_em_ = 570–770 nm, with no significant difference of the emission intensity at *λ*_em_ = 390–570 nm.

To probe the upconversion process, emission spectra were recorded under different excitation powers at *λ*_ex_ = 980 nm (Fig. S10). The dependence of green/red emission intensities on the excitation power was fitted,^39,50^ revealing the number of participating photons of 1.8 and 2.2 for green and red emissions, respectively, for UCNP@BDP-1, which are higher than those of 1.4 and 1.8 for UCNP@IBDP-1, and 1.6 and 1.8 for UCNPs (Fig. S10d and S10e). These results are consistent with the upconversion study and UCQY measurement results, which confirmed the enhanced upconversion emission properties resulting from the photosensitization process from the lanthanide atoms to surface conjugated BDP-1, and an increase of the electron population at the emissive energy level for Er^3+^. The decrease of photons in green and red emissive states of Er^3+^ in UCNP@IBDP-1 nanohybrids is indicative of confined FRET and BET processes,^51^ which lead to weaker upconversion emission and lower values of UCQY. However, the UCQY of UCNP@IBDP-1 measured in the range from 390 nm to 570 nm is higher than that of the UCNPs, which can be attributed to the fluorescence compensation phenomenon and emission of IBDP-1.

The upconversion emission decay curves were recorded to investigate the influence of the BDPs on the lifetime of the Er emission band at *λ*_em_ = 655 nm, corresponding to the ^4^F_9/2_ → ^4^I_15/2_ in Er. For UCNP@BDP-1 and UCNP@IBDP-1 the emission decay was *τ* = 365.2 μs and 280.9 μs, respectively, compared to *τ* = 397.3 μs for OA capped UCNPs (Fig. 3d). The emission lifetime at *λ*_em_ = 540 nm (corresponding to ^4^S_3/2_ → ^4^I_15/2_) was comparable for both nanohybrids with *τ* = 50 μs, which is lower than that of OA capped UCNPs (*τ* = 62 μs) (Fig. S9b). We note that, as expected from UCNPs with high Yb or Er concentrations, there is clear evidence of rise time observed in the lifetime spectra and given the values of 295 μs, 290 μs, and 122 μs for UCNPs, UCNPs@BDP-1, and UCNPs@IBDP-1, respectively (Fig. 3d). The rise time for the 655 nm emissive state was longer than the rise time for the 540 nm emissive state, due to the multiple photon upconversion process.^52^ These results confirm energy transfer between the emissive UCNP (donor) and conjugated BDPs (acceptor), as the BDP absorption band overlaps with the red emission band of Er^3+^. The efficiency of energy transfer between UCNPs and BDPs was estimated as *η* = 1 − *τ*__(UCNP@BDPs)_/*τ*__UCNP_,^38^ and was found to be 8.1% and 29.3% for UCNP@BDP-1 and UCNP@IBDP-1, respectively. We note that the ∼4-times greater energy transfer efficiency of UCNP@IBDP-1 was not accompanied by luminescence quenching (Fig. 3b), which is likely due to the presence of the energy dissipation pathway from the generation of the triplet state in IBDP-1.

The generation of the triplet state of IBDP-1 in UCNP@IBDP-1 nanohybrids under *λ*_ex_ = 980 nm requires the FRET from the UCNPs to BDPs and then intersystem crossing of energy from S_0_ to T_1_ based on the heavy atom effect,^36^ or the direct triplet energy transfer from the lanthanide atom to T_1_ of IBDP-1. To verify which lanthanide atom plays the role in the energy transfer process, we investigated the effect of Er^3+^ doping on the properties of NaYbF_4_:*n*Er^3+^@IBDP-1, with molar ratios *n* from 0.2% to 20%. The XRD and TEM results revealed that the Er^3+^ doped UCNPs have a hexagonal phase and spherical morphology with a comparable size distribution of 20.6 ± 0.9 nm (Fig. S11). The upconversion spectroscopy revealed an increase of upconversion emission intensity with increasing doping only up to 2%Er^3+^ in UCNPs. Further increase of Er^3+^ doping concentration resulted in the decrease of emission intensity (Fig. S12a and b).^34^ For Er^3+^ doped UCNP@IBDP-1, the highest upconversion emission intensity was observed with 0.5% doping. In highly doped systems, Er^3+^ can induce concentration quenching and cross-relaxation^42^ processes between the sensitizer Yb^3+^ and activator Er^3+^. At relatively low doping concentration, Er^3+^ contributes additional energy dissipation pathways in UCNP@BDP-1.

**Fig. 4:**
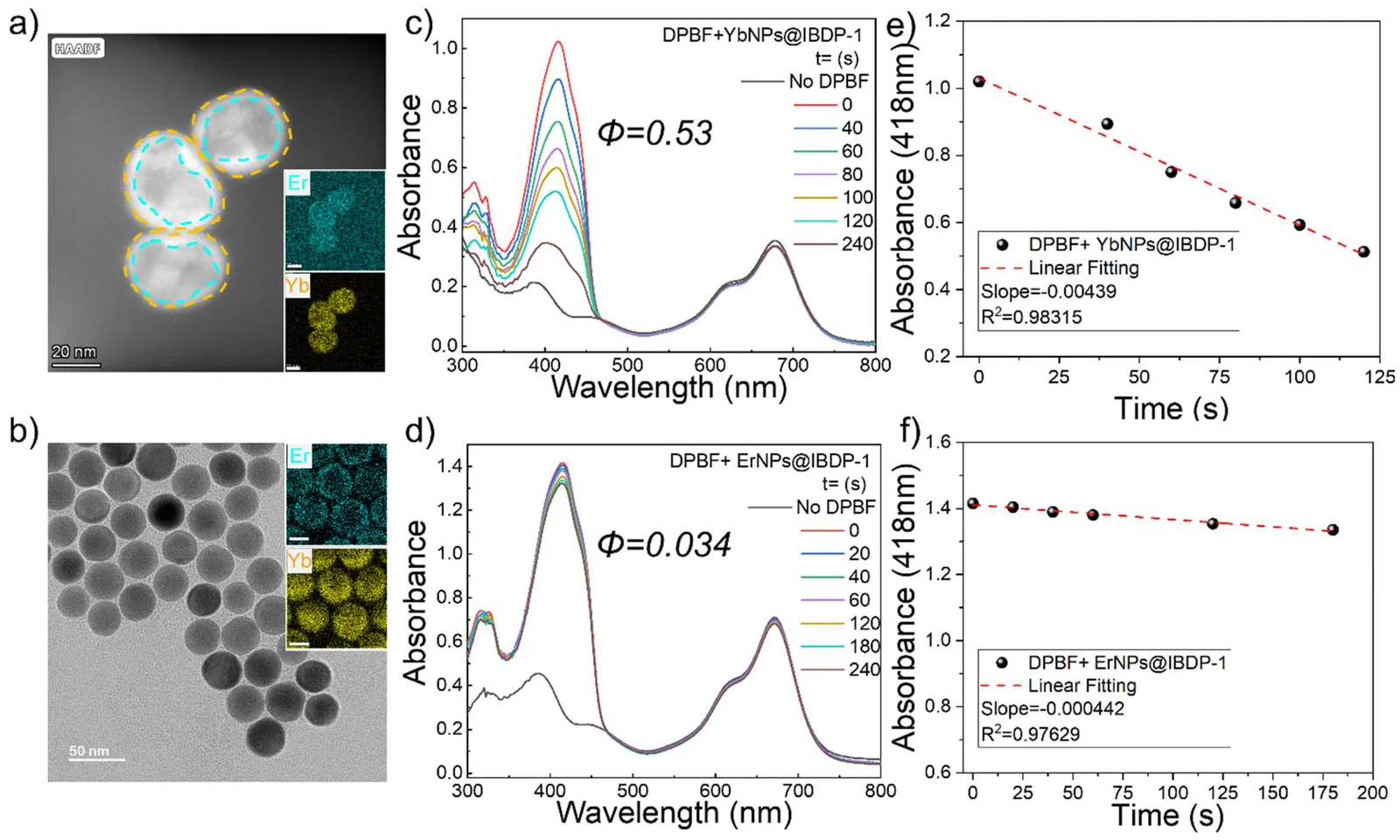
(a) Representative high-angle annular dark-field (HAADF) image of YbNPs@IBDP-1 and (b) TEM image of ErNPs@IBDP-1 and (inset) elemental mapping of Yb and Er; scale bar 20 nm. Absorption spectra of DPBF with (c) YbNPs@IBDP-1 and (d) ErNPs@IBDP-1 recorded at different time points up to *t* = 240 s under continuous irradiation. Absorption intensity of (e) YbNPs@IBDP-1 and (f) ErNPs@IBDP-1 measured at 418 nm under continuous irradiation with *λ*_ex_ = 980 nm for *t* = 180 s and fitted (dashed line).

We further prepared the NaYbF_4_ sensitive shell and NaErF_4_ active shell for NaYbF_4_:2%Er^3+^@IBDP-1, to facilitate IBDP-1 anchoring to Yb^3+^ and Er^3+^, respectively. The core–shell NaYbF_4_:2%Er^3+^@NaYbF_4_@IBDP-1 (YbNP@IBDP-1) and NaYbF_4_:2%Er^3+^@NaErF_4_@IBDP-1 (ErNP@IBDP-1) retained hexagonal phase (Fig. S13a) spherical nanoparticles, with elemental mapping revealing a clear core–shell structure (Fig. 4a and d). The shell thickness was estimated to be 1.8 ± 0.9 nm and 1.9 ± 0.3 nm for YbNP@IBDP-1 and ErNP@IBDP-1, respectively (Fig. S13).

The efficiency of triplet state generation of IBDP-1 was evaluated indirectly by recording the singlet oxygen (^1^O_2_) induced absorption decay of 1,3-diphenylisobenzofuran (DPBF).^13,38^ DMSO solutions containing different amounts of DPBF were prepared and Rose Bengal (RB) was used as a reference ^1^O_2_ generator. Under irradiation with a 980 nm (8 W cm^−2^) laser, the absorbance of DPBF at 418 nm was recorded and used to calculate the ^1^O_2_ yield, *ϕ*, as^13^1
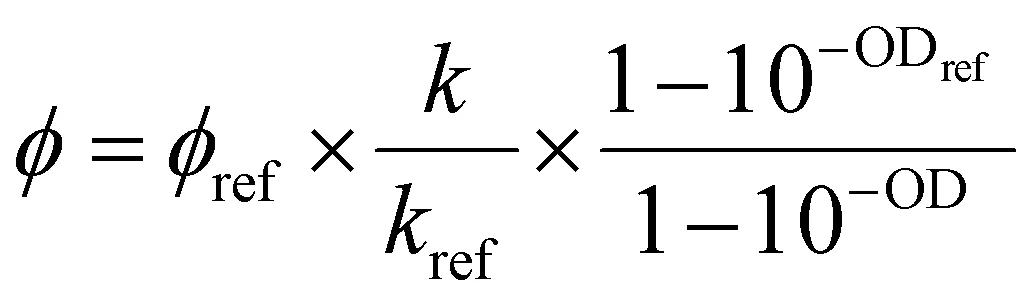
where *ϕ*_ref_ is the ^1^O_2_ yield of the reference RB sample; *k* and *k*_ref_ are the slopes of the decrease of the absorbance at 418 nm measured with a sensitizer and reference RB, respectively; and OD and OD_ref_ are the measured absorbance values of the sensitizer and reference RB, respectively.

For YbNP@IBDP-1 with DPBF the absorbance at 418 nm decreased linearly (*R*^2^ ∼ 0.98) by a factor of two with increasing irradiation time up to 2 min (Fig. 4c and e). However, ErNP@IBDP-1 showed a small (∼10%) decrease of absorbance with the same irradiation protocols (Fig. 4d and f). The ^1^O_2_ yield of YbNP@IBDP-1 was calculated as *ϕ* = 0.53, which was ∼16× higher than that of ErNP@IBDP-1, suggesting higher triplet generation efficiency of Yb atoms to IBDP-1 in YbNP@IBDP-1 compared to that of Er atoms in ErNP@IBDP-1. We note that the measured low ^1^O_2_ yields when a sample of the YbNPs and IBDP-1 were mixed with *ϕ* = 0.05 (Fig. S14c) are as expected for long distance energy transfer and the re-absorption process. These results revealed that a triplet energy transfer route in YbNP@IBDP-1, and high triplet yield were achieved through the Yb^3+^ from the sensitive shell to anchored IBDP-1. In contrast, the unmeasurable ^1^O_2_ yields of ErNP@IBDP-1 indicated the blocking of the triplet energy transfer route by the presence of the NaErF_4_ shell. We note that a 1.9 nm thick NaErF_4_ shell is still within the FRET distance, and the FRET dominated the energy transfer in ErNP@IBDP-1.

**Fig. 5:**
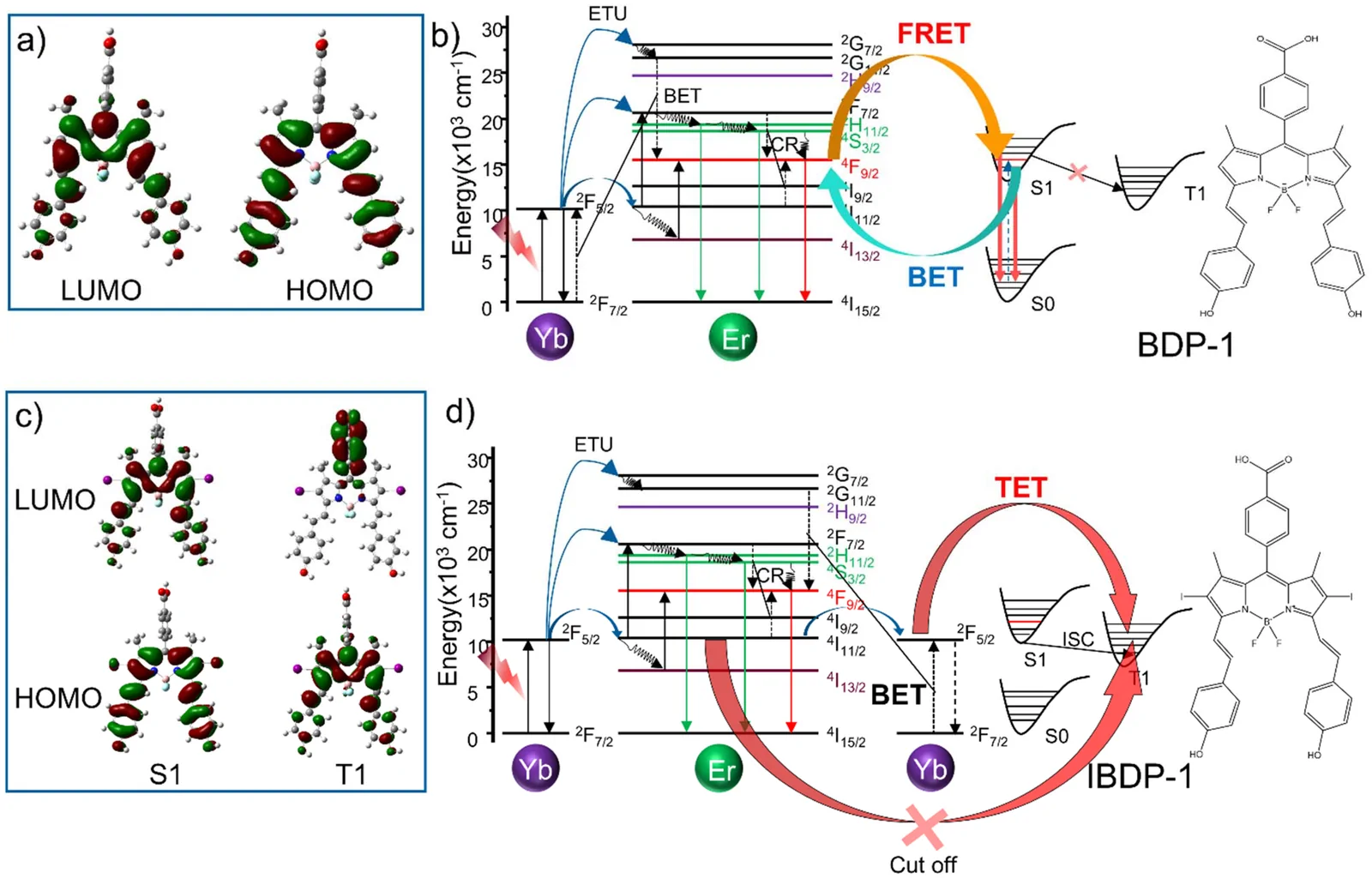
Schematic diagrams of energy transfer mechanisms between BDPs and UCNPs under NIR excitation. (a) Molecular frontier orbital maps of BDP-1 and (b) FRET from UCNP to BDP-1. (c) Molecular frontier orbital maps of IBDP-1 and (d) TET for generating a triplet.

Time-dependent density functional theory (TD-DFT) quantum chemistry calculations were performed to explore the mechanism of energy transfer in these UCNP@BDP nanohybrids. Calculations showed that the electrons on the highest occupied molecular orbital (HOMO) and the lowest unoccupied molecular orbital (LUMO) of BDP-1 were mainly located in the core of the BDP and extended chains (Fig. 5a), which suggested that Förster resonance energy transfer (FRET) occurred to produce the fluorescence compensation,^53^ and the backward energy transfer (BET) took place from S_1_ of BDP-1 (donor) to the emissive energy level of Er^3+^ (acceptor), which increased the populating photons and benefited the emission (Fig. 5b). We note that the BET process exhibits distance-dependent behaviour, consistent with the FRET mechanism. The electrons on the HOMO/LUMO of the first singlet of IBDP-1 were similar to those of BDP-1 (Fig. 5c). However, the heavy atom effect of I enables IBDP-1 to generate the triplet excited state, and affect the electrons in the HOMO/LUMO state of the first IBDP-1 triplet. The electron in the HOMO is mainly located in the core of the BDP and extended chains, but the electrons in the LUMO are mainly located on the benzene ring and carboxyl group (Fig. 5c). These results further evidence the direct triplet energy transfer (TET) process for IBDP-1 anchored to the Yb on the surface of UCNPs through the carboxyl group.

The possible energy transfer mechanism can be summarized as follows: upon 980 nm laser irradiation, Yb can absorb the NIR photons and excite Er to ^2^G_7/2_ through the multiple energy transfer upconversion (ETU) process. In BDP-1 modified nanohybrids (Fig. 5b), the FRET can be achieved from the ^4^F_9/2_ in Er^3+^ to the S_1_ of anchored molecules (LUMO), by matching the emissive energy levels of lanthanide ions to the absorption of the organic molecules. The BET process^39^ can occur from the S_1_ of the molecules (HOMO) to the ^4^F_9/2_ in Er^3+^, and results in enhanced upconversion emission of Er^3+^ in UCNP@BDP-1 nanohybrids. Note that a proportion of energy in S_1_ can transfer back to S_0_ and resulting in the fluorescence compensation phenomenon. In IBDP-1 modified nanohybrids (Fig. 5d), the TET process from ^2^F_5/2_ of Yb^3+^ to T_1_ of IBDP-1 was achieved by anchoring IBDP-1 to Yb^3+^. We note that TDDFT revealed that both ^2^F_5/2_ in Yb^3+^ and ^4^I_11/2_ in Er^3+^ were matched to the energy of T_1_ in IBDP-1 (1.01 eV), with higher efficiency TET observed for Yb^3+^-IBDP_1 compared to that for Er^3+^-IBDP_1 (Fig. 4), likely due to the difference of the spin-exchange coupling with the unpaired electrons in the Ln^3+^ ions to the excited state in the organic component. The high efficiency of the TET process can skip the traditional FRET-ISC process and enable the generation of the ^1^O_2_ or other photocatalytic reactions.

## 4. Conclusions

In this work we presented boron dipyrromethene (BDP-1 and IBDP-1) modified NaYbF_4_ based upconversion nanohybrids with enhanced upconversion properties under NIR illumination, and demonstrated that the FRET and BET mechanisms are responsible for the observed properties. Furthermore, UCNP@IBDP-1 nanohybrids were found to be an effective ^1^O_2_ generation agent with a yield of 0.53 under NIR irradiation, and the triplet generation was selective to the lanthanide atoms. Combined with the TDDFT calculations of molecular frontier orbital analysis, the direct triplet energy transfer mechanism from the Yb to T_1_ of anchored IBDP-1 molecules was proposed. These results provide a strategy for the selection of BDP molecules and specific lanthanide atoms in the UCNP@BDP nanohybrids to tailor the upconversion performance or triplet generation ability. These findings advance our understanding of the nature of energy transfer in organic–inorganic nanohybrids, and pave the way for constructing bright fluorescent probes, photodynamic therapy or other photocatalytic applications.

## Author contributions

WX and YW conducted all experiments and modelling. All co-authors contributed to data analysis and interpretation, co-wrote the manuscript and approved its submission.

## Conflicts of interest

All authors declare no conflicts of interest.

## Supplementary Material

NR-018-D6NR02161A-s001

## Data Availability

The data that support the findings of this study are available from the corresponding author upon reasonable request. Supplementary information (SI) is available. The data supporting this article include NMR and HRMS, UV–Vis absorption spectra, XRD, TEM, FRET, DLS, upconversion emission/decay spectra, and calculated energy states of BDP-1/IBDP-1. See DOI: https://doi.org/10.1039/d6nr02161a.
