# Supplementary material for: Tailoring triplet energy transfer and Förster resonance energy transfer in boron dipyrromethene modified lanthanide doped upconversion nanohybrids
Source: Nanoscale. 2026 Aug 19;18(35):19132–42. doi: 10.1039/d6nr02161a (PMC13583994; doi:10.1039/d6nr02161a)
Supplement: Supplementary file 1 [file NR-018-D6NR02161A-s001.pdf]

Supplementary Information (SI) for Nanoscale.
This journal is © The Royal Society of Chemistry 2026

Supporting Information

Tailoring Triplet Energy Transfer and Förster resonance energy transfer in the Boron Dipyrromethene Modified Lanthanide doped Upconversion Nanohybrids

Weitong Xiao,[a,b] *Yiting Wu,*[a] *Feiran Wang,*[b] *Lyudmila Turyanska,*[b]*
Huimei Yu,[c] *and Weiling Luan* [a]*

[a] CPCIF Key Laboratory of Advanced Battery Systems and Safety, School of Mechanical and Power Engineering, East China University of Science and Technology, Shanghai 200237, China

[b] Centre for Additive Manufacturing, Faculty of Engineering, University of Nottingham, Jubilee Campus, Nottingham, NG8 1BB, UK

[c] School of Materials Science and Engineering, East China University of Science and Technology, Shanghai 200237, China

*Corresponding address: Luan@ecust.edu.cn; Lyudmila.Turyanska@nottingham.ac.uk

SI1. Supporting results

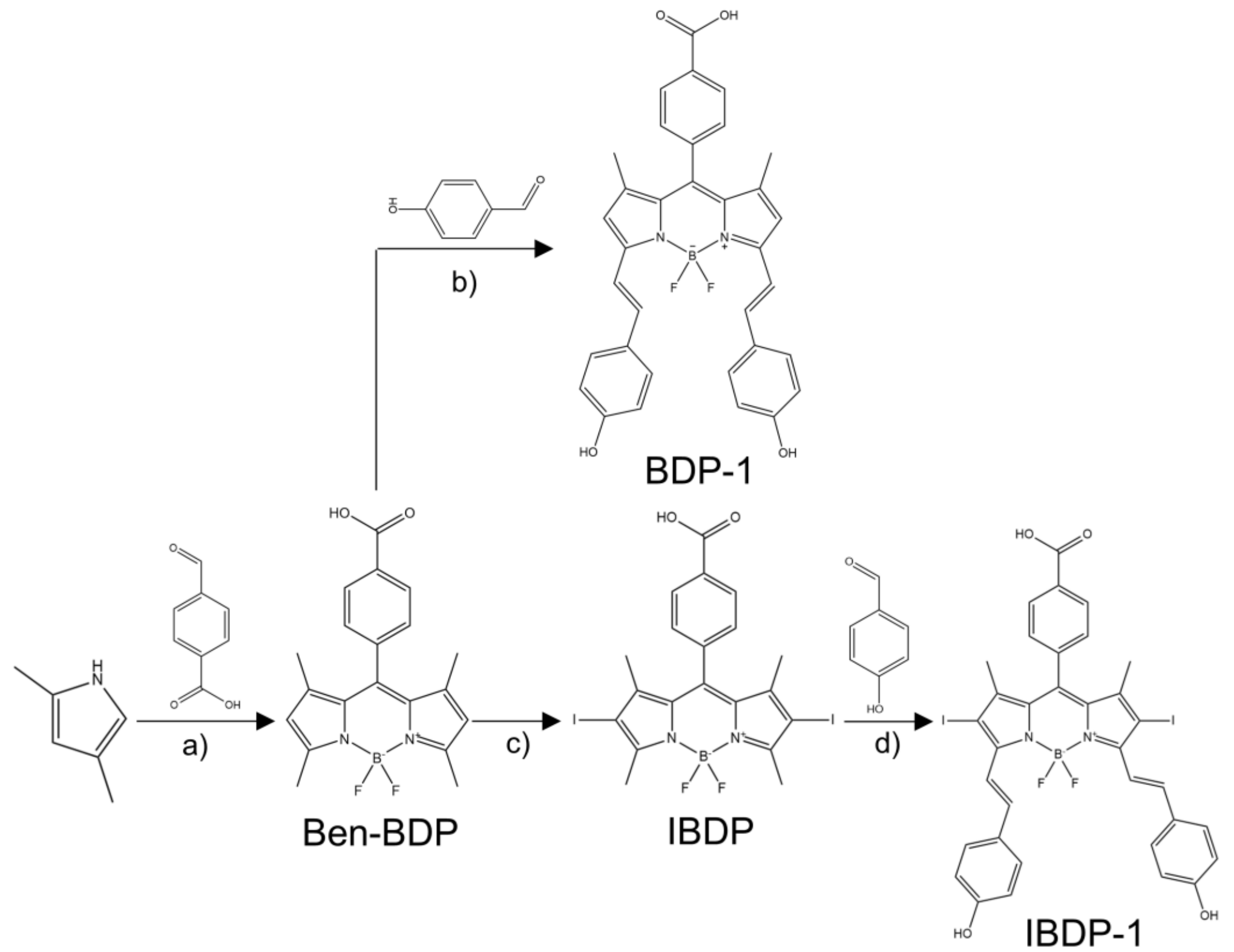

Scheme S1. Scheme of the synthesis of BDPs. **a)** i: CH_2Cl_2, 6h, room temperature; ii: DDQ, 1h; iii: Et_3N, BF_3OEt_2, 12h. **b)** Toluene, piperidine, AcOH, 130°C, refluxing. **c)** EtOH, iodine, iodic acid, H_2O, 60°C, 2h. **d)** Toluene, piperidine, AcOH, 130°C, refluxing.

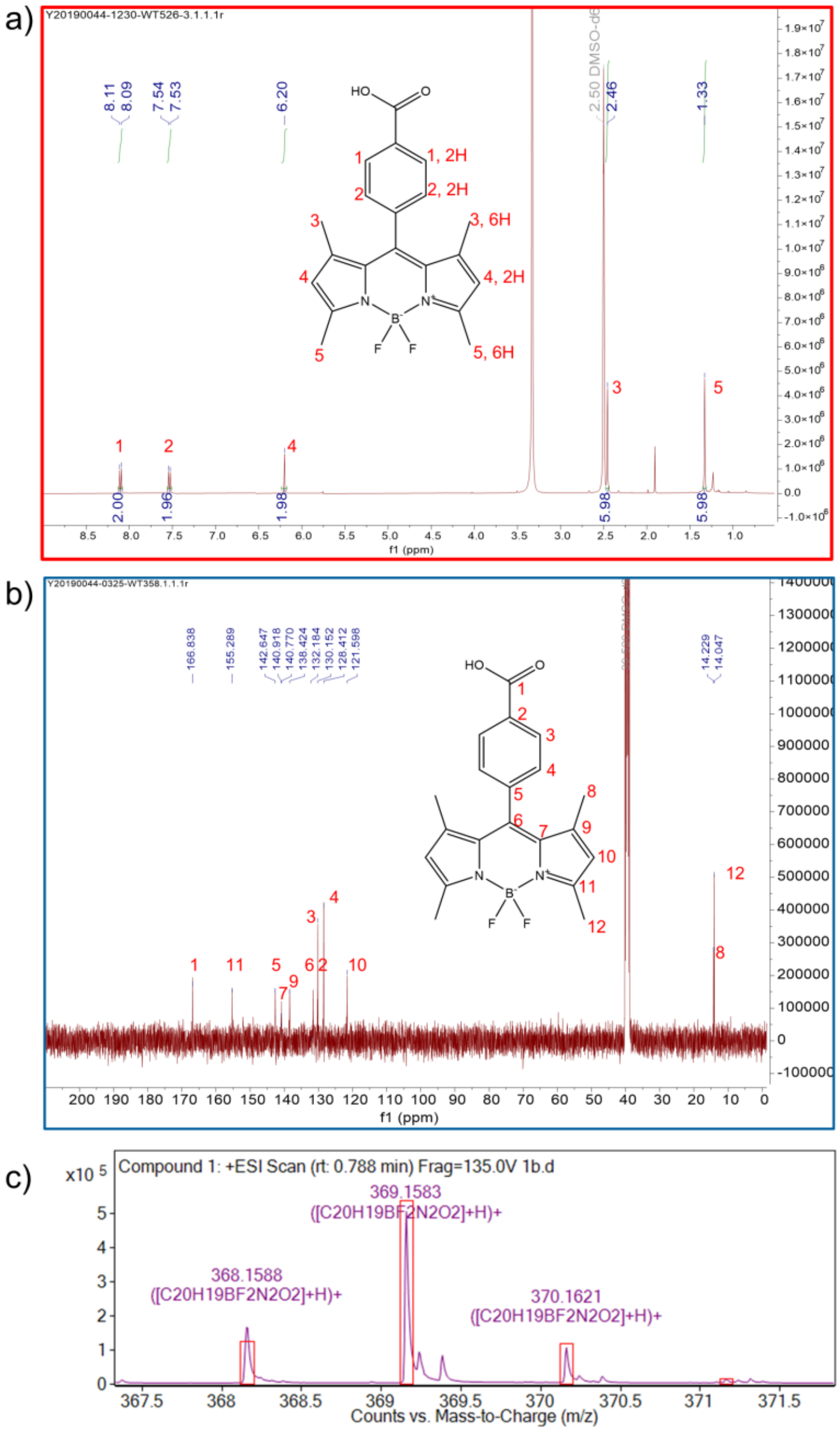

Figure S1 ^{1}H-NMR **(a)**, ^{13}C-NMR **(b)** and HRMS **(c)** spectra of the Ben-BDP.

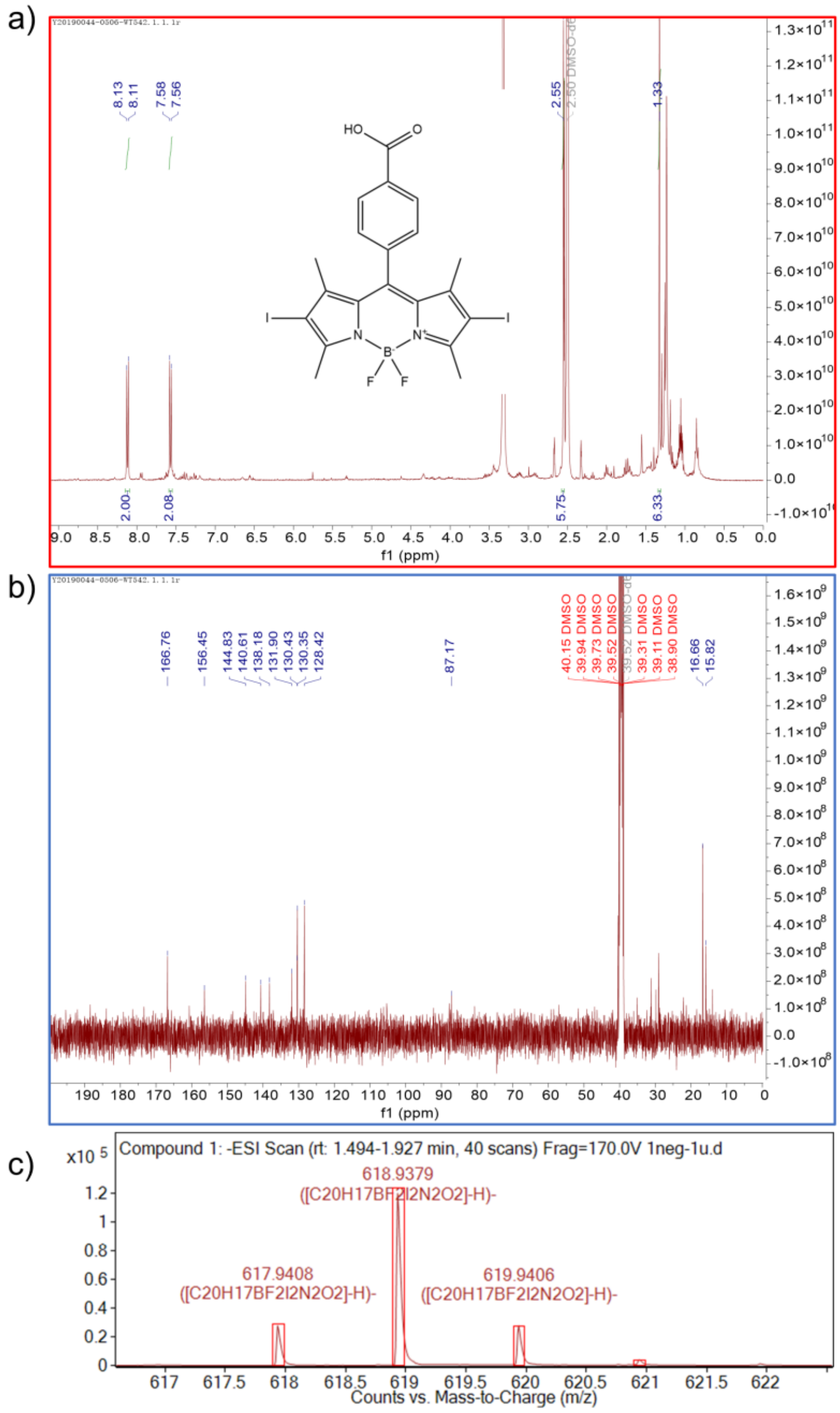

Figure S2 ^{1}H-NMR **(a)**, ^{13}C-NMR **(b)** and HRMS **(c)** spectra of the IBDP.

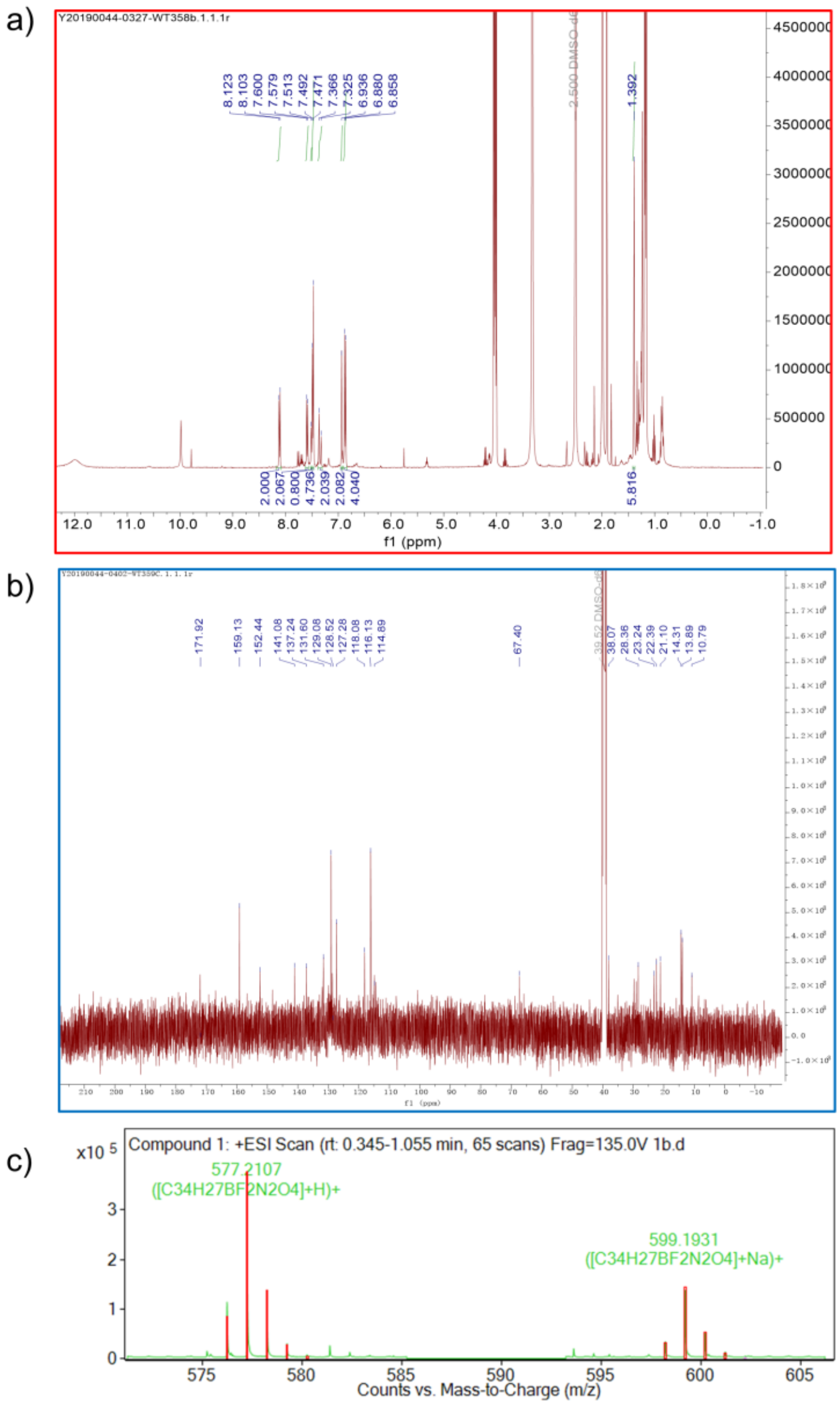

Figure S3 ^{1}H-NMR **(a)**, ^{13}C-NMR **(b)** and HRMS **(c)** spectra of the BDP-1.

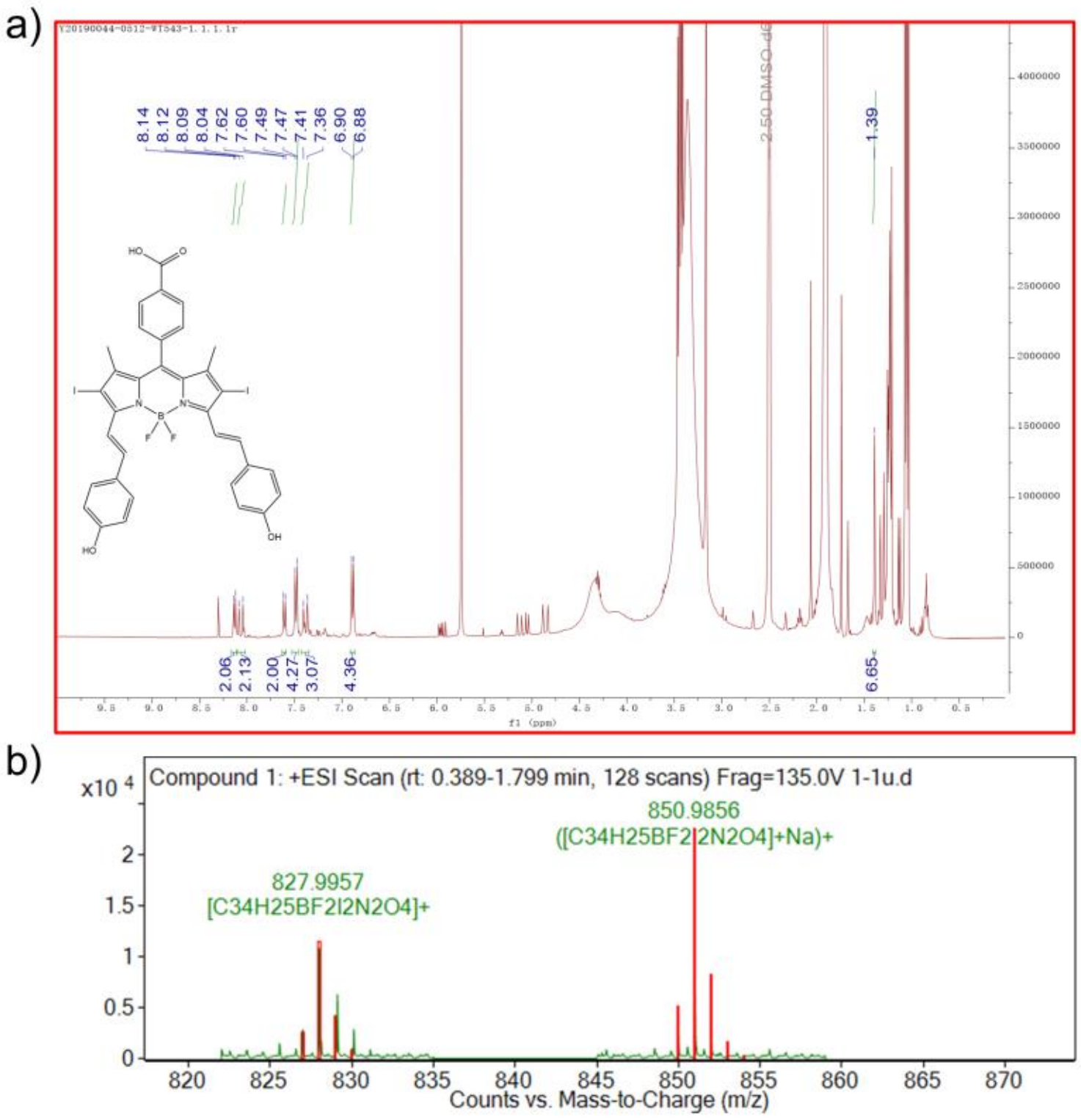

Figure S4 ^{1}H-NMR **(a)** and HRMS **(b)** spectra of the BDP-1.

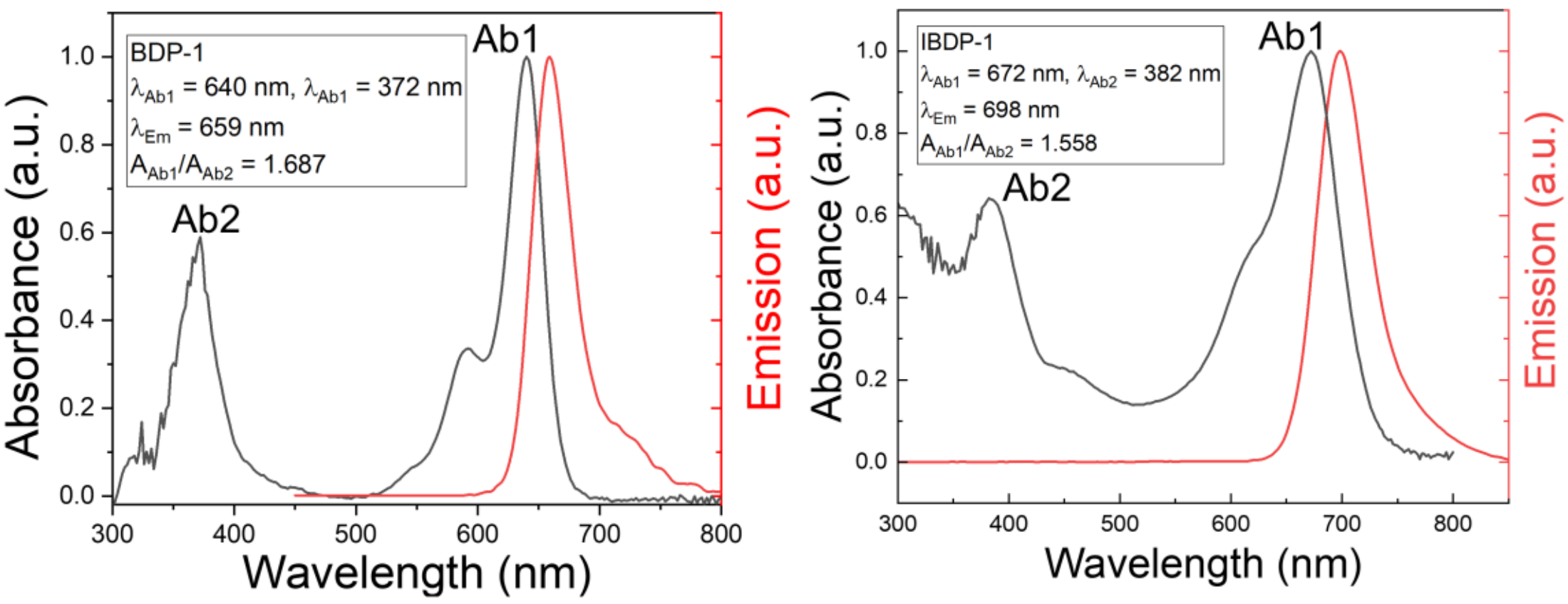

Figure S5 Absorption spectra of the pure BDP-1 (left) and IBDP-1 (right) from 300 nm to 800 nm in DMSO. The peaks positions were shifted from 640 nm of pure BDP-1 to 653 nm of the UCNP@BDP-1 nanohybrids for first absorption peak, and 372 nm of pure BDP-1 shifted to 379 nm of the UCNP@BDP-1 nanohybrids for second absorption peak. The ratio of the absorbance of the first absorption peak to second absorption peak was decrease from 1.687 (Pure BDP-1) to the 1.347 (UCNP@BDP-1). Similarly, these changes were also observed in the pure IBDP-1 and UCNP@IBDP-1 nanohybrids. Interestingly, the peak position of the first absorption kept consistent at 672 nm, but the ratio of the absorbance of the first absorption peak to second absorption peak was decrease significantly from 1.558 to 0.221.

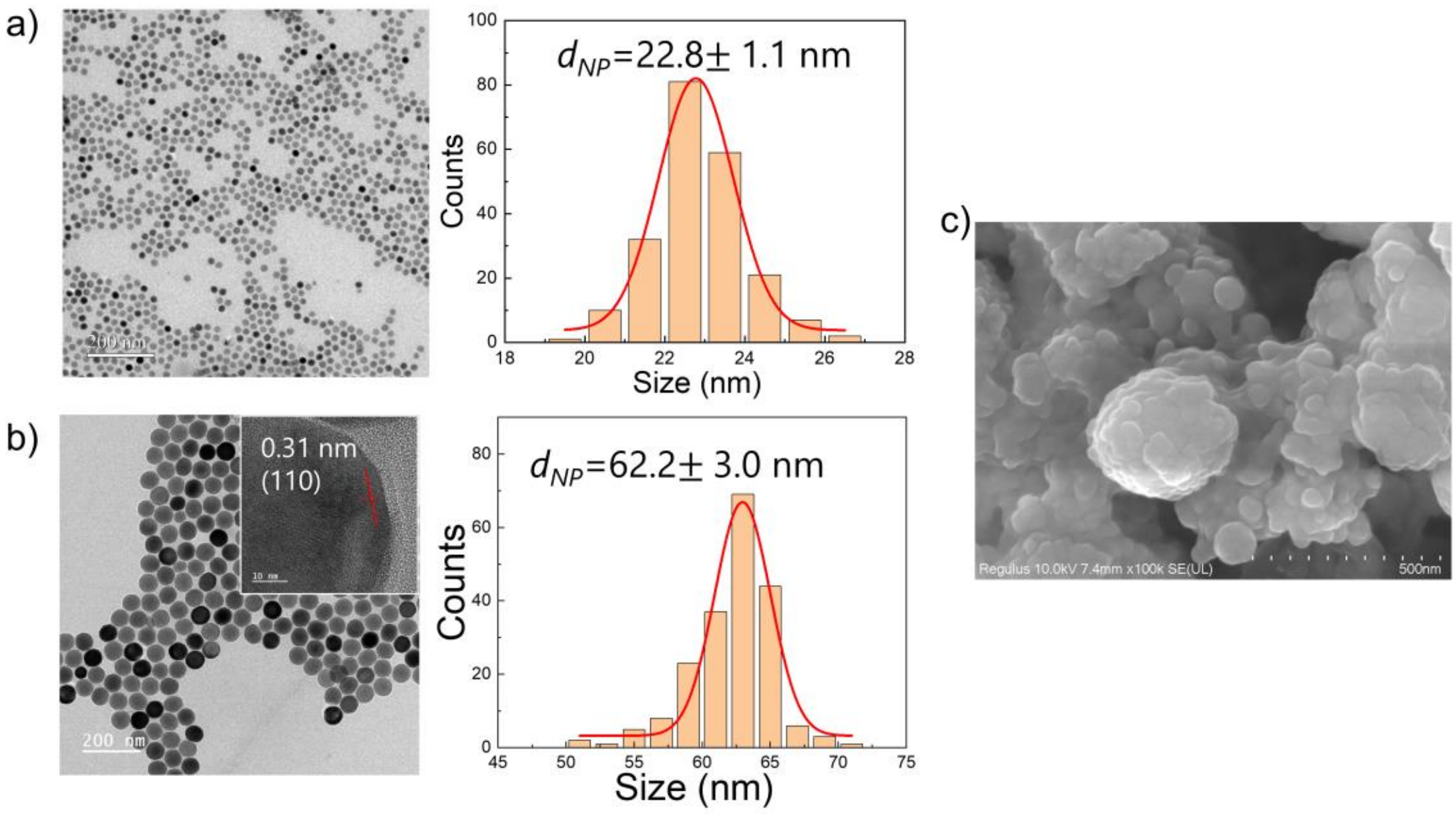

Figure S6 Representative TEM/HR-TEM images and size distributions of the final products obtained by **(a)** IM protocol and **(b)** SA protocol. **c)** the SEM images of the intermediates obtained by the IM protocol.

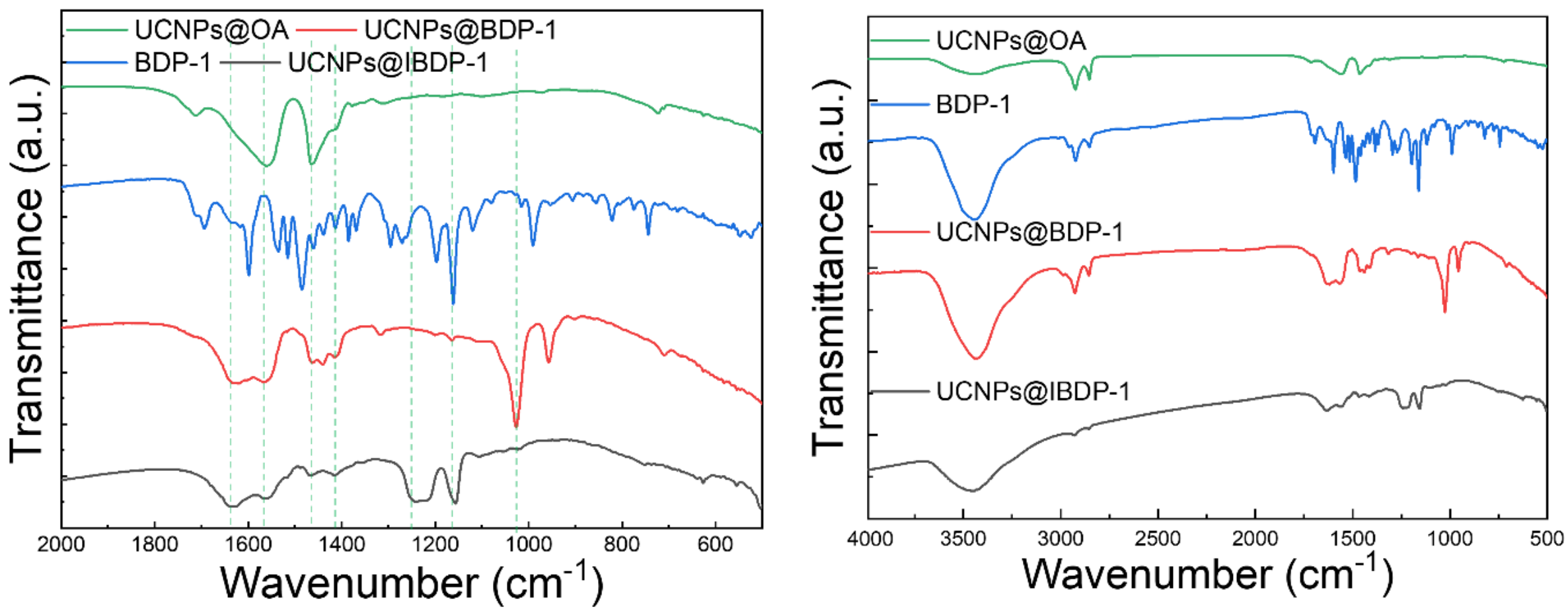

Figure S7 FTIR spectra of the as-synthesized UCNP@BDPs nanohybrids.

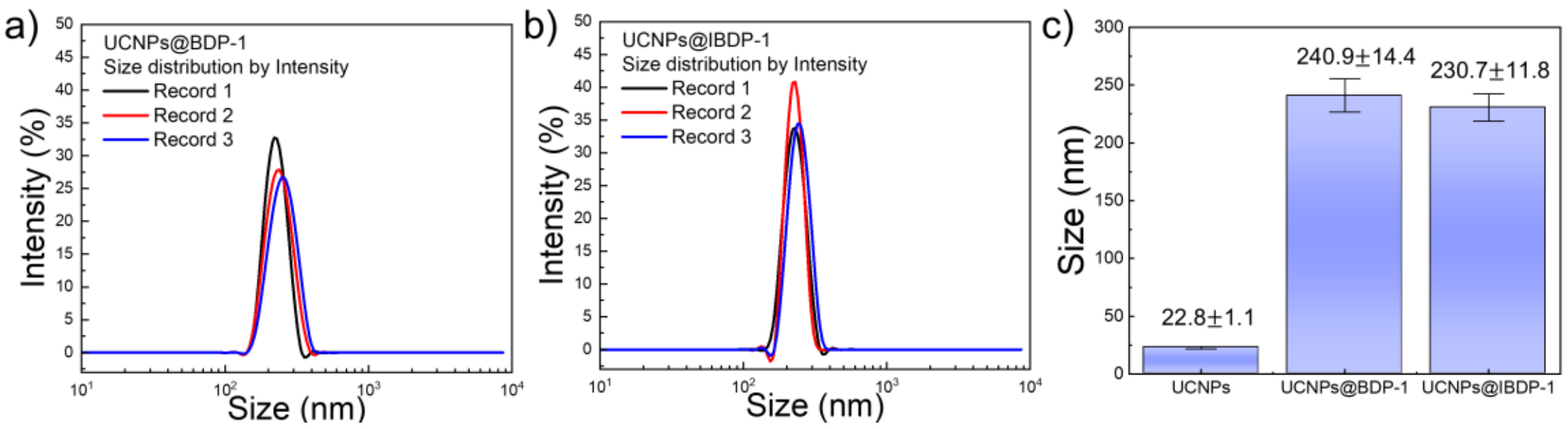

Figure S8. DLS size distributions of of the as-synthesized **(a)** UCNP@BDP-1 and **(b)** UCNP@IBDP-1 nanohybrids. **(c)** Hystograme of the average sizes of original UCNPs estimated from TEM (NPs dispersed in hexane) and hydrodynamic size of the UCNP@BDP nanohybrids measured by DLS in ultra-pure water with a concentration of 1 mg/ml.

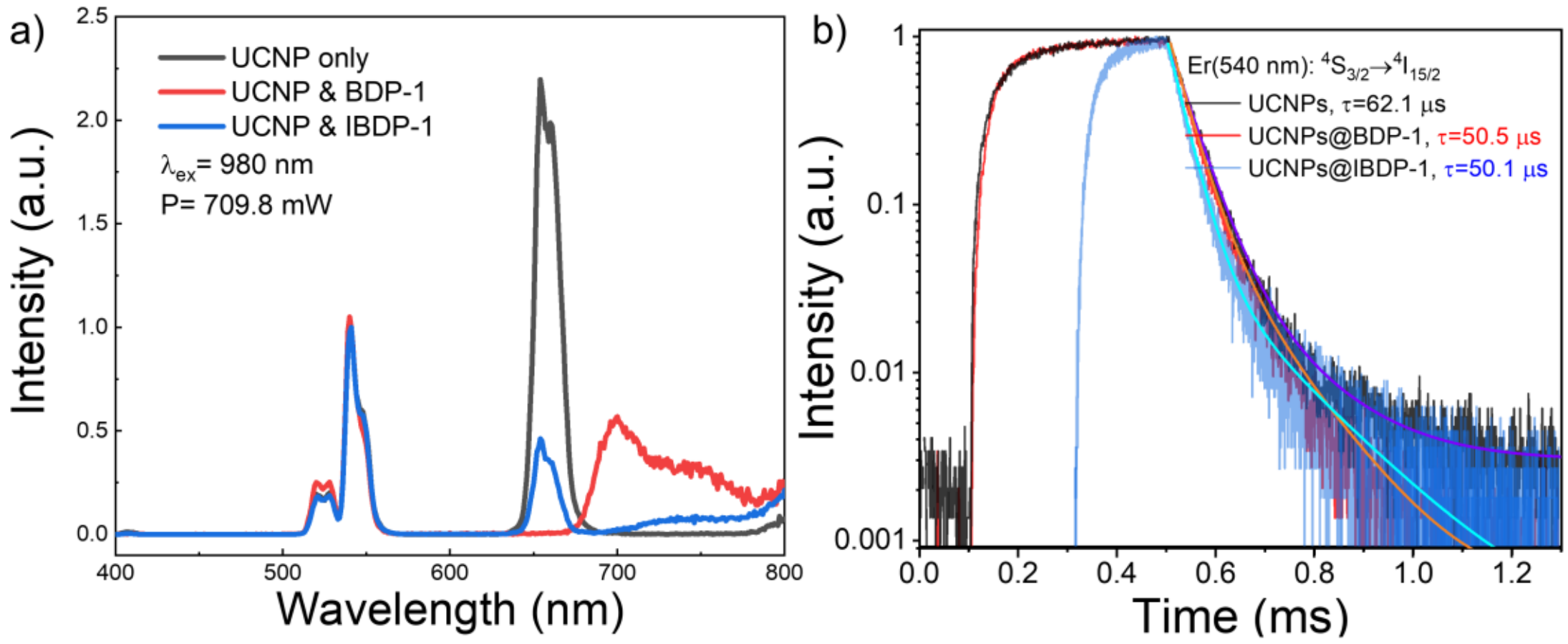

Figure S9 a) Upconversion spectra of the simply mixing (SM) samples of the UCNP and BDPs under 980 nm excitation in DMSO solution. Upconversion emission intensity was normalized to 540 nm, where no absorption band of BDPs was observed and no interaction between BDPs and UCNPs. The spectra indicated the quenching of the emission of Er^{3+} at λ_{em} = 655 nm, and new emission features showed at λ_{em} = 700 nm and 740 nm. **b)** Upconversion emission decay of the UCNP and UCNP@BDPs nanohybrids at λ_{em} = 540 nm. The decay of the UCNPs@BDP-1 showed no difference to the UCNPs@IBDP-1. The risetime calculations gave the value of 191 μs, 195 μs, 120 μs for UCNPs, UCNPs@BDP-1, UCNPs@IBDP-1, respectively.

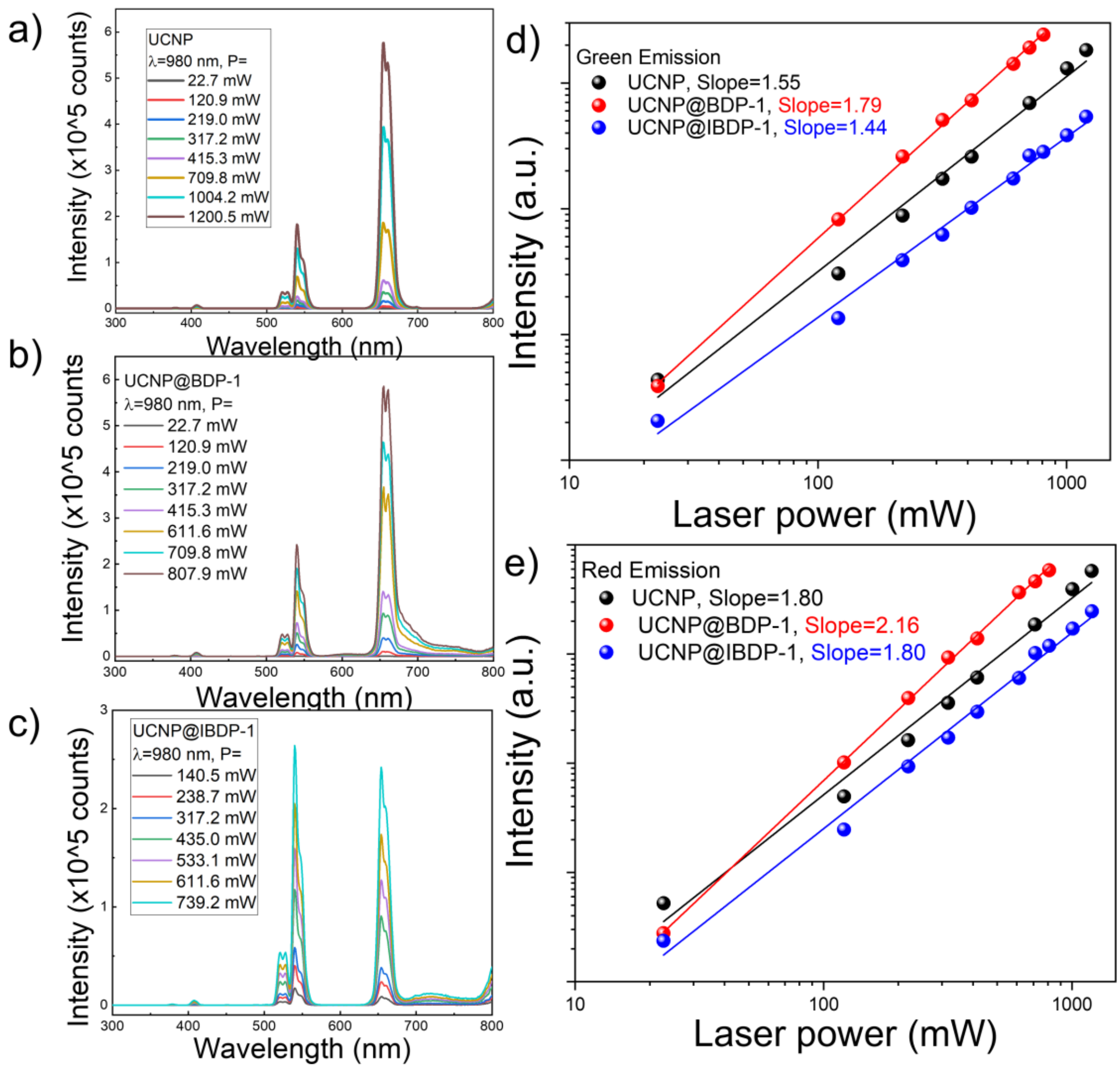

Figure S10 Upconversion emission intensity of **a)** UCNPs, **b)** UCNP@BDP-1 and **c)** UCNP@IBDP-1 under different excitation power at λ_{ex} = 980 nm. Upconversion emission intensity of **d)** green emission (λ_{em} = 540 nm) and **e)** red emission (λ_{em} = 655 nm) as a function of the excitation power density, linear fitting was applied and the slope was related to the number of participating photons in Er^{3+}. The participating photons increase for the BDP-1 modified nanohybrids, but no change or even decrease for IBDP-1 modified nanohybrids.

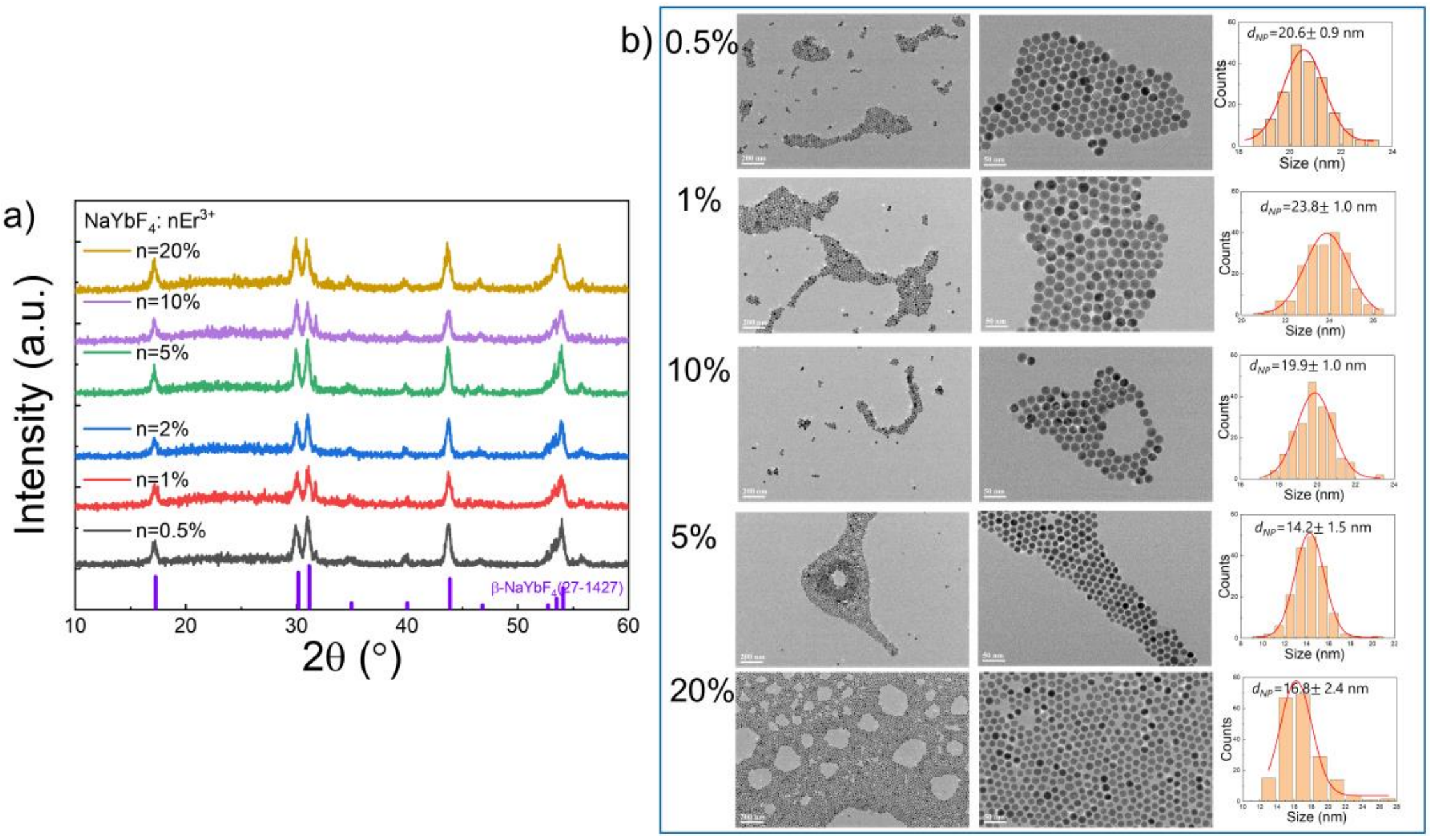

Figure S11 a) XRD analysis of the samples produced with different Er^{3+} doping concentration, n, in the $NaYbF_4$. **b)** TEM images and corresponding size distributions of $NaYbF_4$: nEr^{3+}.

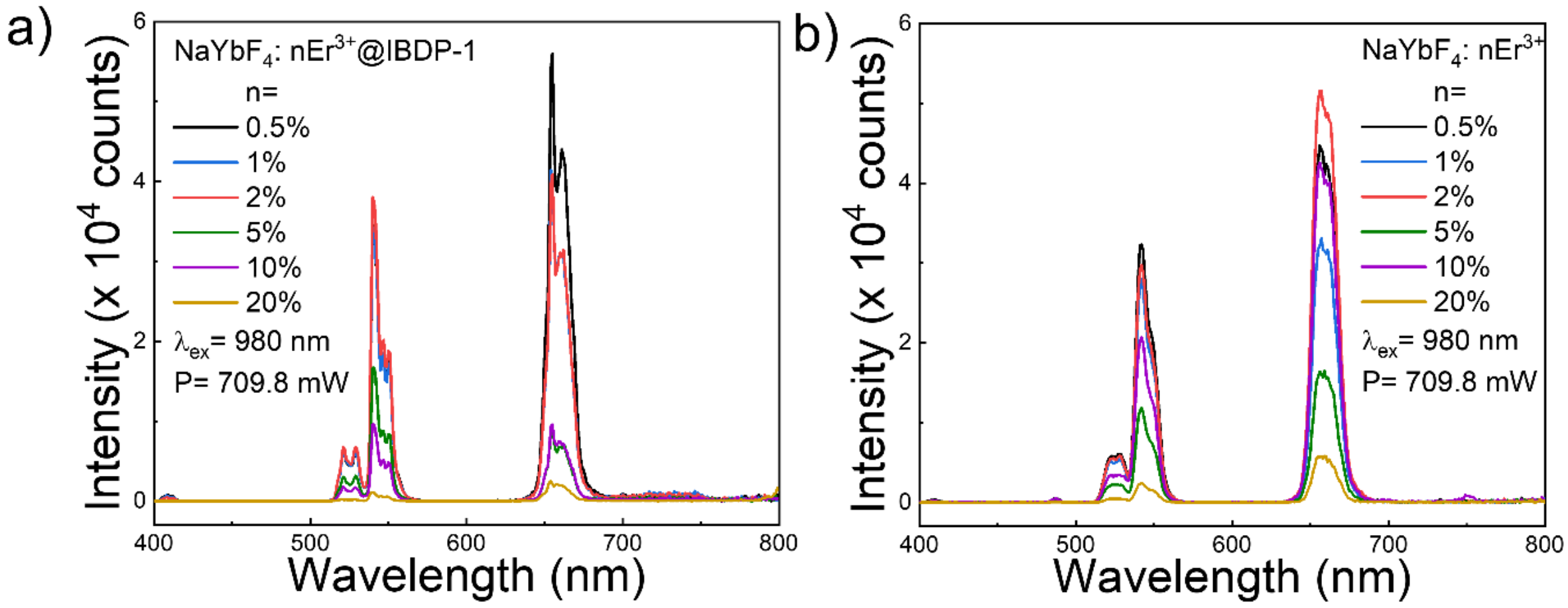

Figure S12 Upconversion spectra of **a)** $NaYbF_4$: nEr^{3+} @IBDP-1 and **b)** $NaYbF_4$: nEr^{3+} under λ_{ex} = 980 nm.

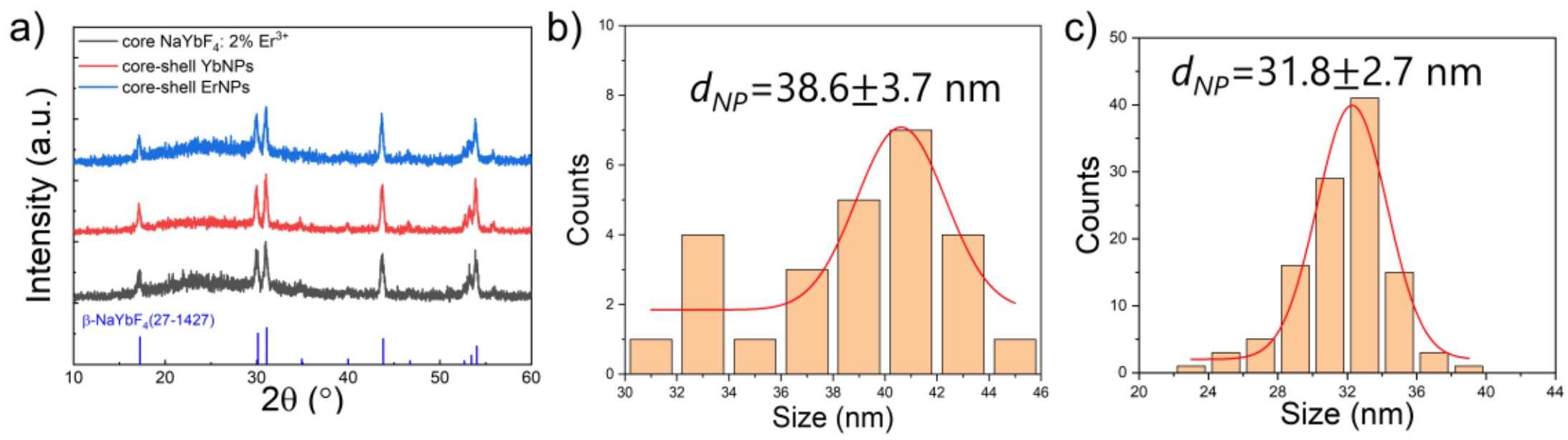

Figure S13 a) the XRD analysis of the core and core-shell UCNPs. The size distribution of the **b)** YbNP@IBDP-1 and **c)** ErNP@IBDP-1.

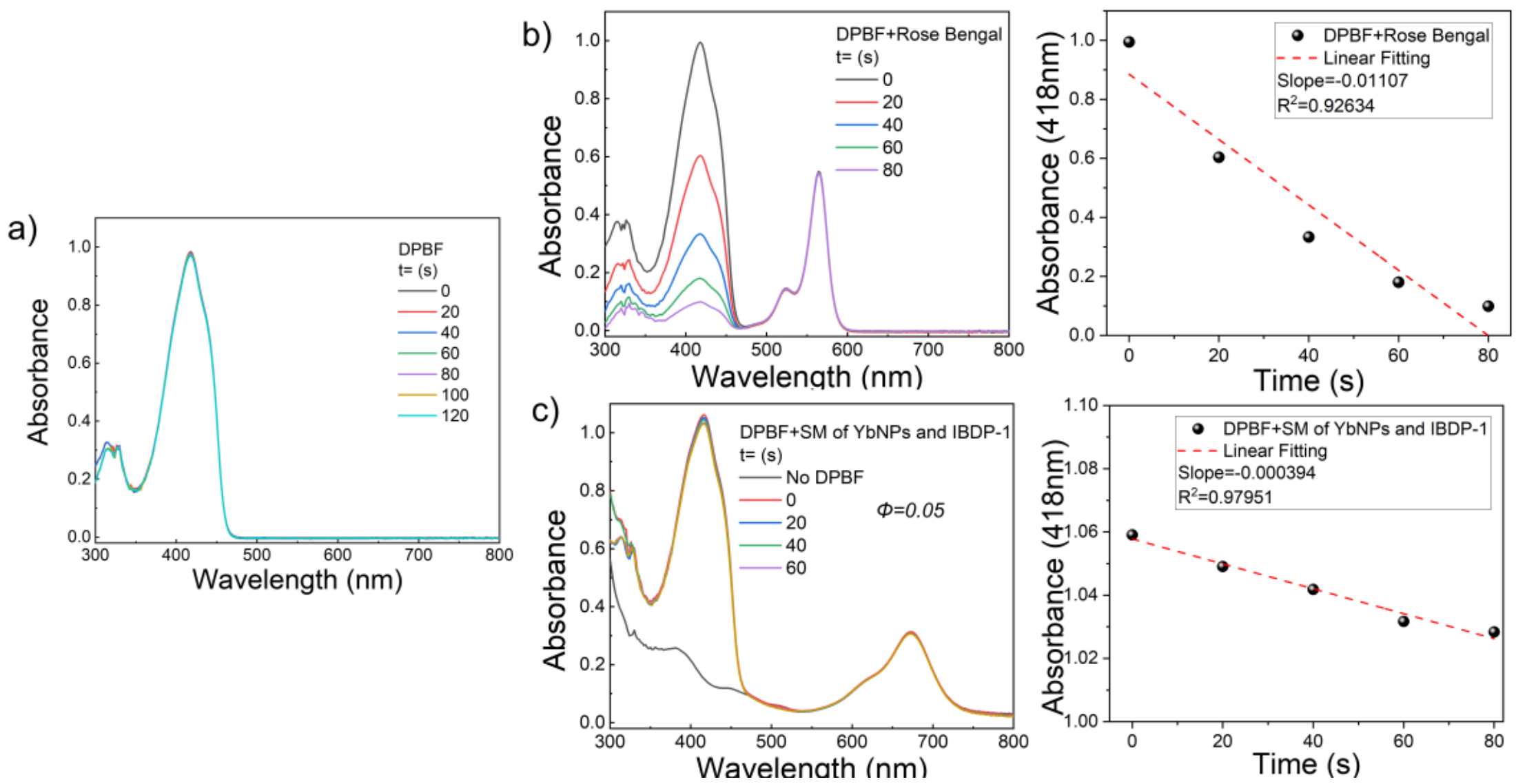

Figure S14 $^1O_2$ release measurement of **a)** no PS involved, **b)** using Rose Bengal as reference and **c)** simply mixing sample of YbNP and IBDP-1.

SI2. Supporting data.

The excitation states, energies, orbitals and oscillator strengths of the BDPs were calculated by TDDFT and list below. Note, the geometry optimization of the molecule was performed using DFT before TDDDT.

First three singlets for BDP-1:

```
Excited State   1:   Singlet-A   2.1559 eV 575.10 nm f=0.8839 <S**2>=0.000
     150 ->151        0.69131

Excited State   2:   Singlet-A   3.4851 eV 355.76 nm f=1.4143 <S**2>=0.000
     141 ->151        0.11378
     149 ->151        0.67761

Excited State   3:   Singlet-A   3.8010 eV 326.19 nm f=0.0309 <S**2>=0.000
     147 ->151        0.67108
     148 ->151       -0.13404
```

First three singlets for IBDP-1:

```
Excited State   1:   Singlet-A   1.8292 eV 677.80 nm f=0.7710 <S**2>=0.000
     156 ->157        0.70855

Excited State   2:   Singlet-A   2.5855 eV 479.53 nm f=0.5425 <S**2>=0.000
     155 ->157        0.68537
     156 ->159        0.16565

Excited State   3:   Singlet-A   2.8377 eV 436.92 nm f=0.0049 <S**2>=0.000
     152 ->157        0.17287
     154 ->157        0.68104
```

First three triplets for IBDP-1:

```
Excited State   1:   Triplet-A   1.0112 eV 1226.15 nm f=0.0000 <S**2>=2.000
     152 ->157       -0.11565
     156 ->157        0.70130
     156 <-157        0.11948

Excited State   2:   Triplet-A   1.9530 eV 634.84 nm f=0.0000 <S**2>=2.000
     145 ->157        0.11945
     155 ->157        0.64522
     156 ->159       -0.21655

Excited State   3:   Triplet-A   2.4501 eV 506.03 nm f=0.0000 <S**2>=2.000
     152 ->157        0.17911
     154 ->157        0.63859
     155 ->159        0.12425
     156 ->160        0.13134
```
